# Coupling antigorite deformation and dehydration in high-pressure experiments

**DOI:** 10.1007/s00410-025-02255-z

**Published:** 2025-08-23

**Authors:** Lisa Eberhard, Mattia Luca Mazzucchelli, Stefan Markus Schmalholz, Holger Stünitz, Ahmed Addad, Patrick Cordier, Oliver Plümper

**Affiliations:** 1 Department of Earth Sciences, Utrecht University, Utrecht, The Netherlands; 2 Institute of Earth Sciences, University of Lausanne, Lausanne, Switzerland; 3 Institut des Sciences de la Terre d’Orléans, Université d’Orléans, Orléans, France; 4 Department of Geosciences, University of Tromsø, Tromsø, Norway; 5 University of Lille, CNRS, INRAE, Centrale Lille, UMR 8207, UMET, Unité Matériaux et Transformations, Lille, France; 6 Institut universitaire de France (IUF), Paris, France; 7 Fachbereich Geowissenschaften, University of Bremen, Bremen, Germany

**Keywords:** Serpentine dehydration, Deformation, Griggs experiment, Reaction kinetics, Mechanical work rate, Hydro-mechanical-chemical HMC coupling

## Abstract

The dehydration of antigorite is an important reaction in subduction zones with implications on both geochemical and geophysical processes. In this experimental study we focus on the onset of antigorite dehydration and investigate various chemical and physical parameters as possible drivers for the fluid release. We performed hydrostatic and co-axial Griggs experiments on antigorite serpentinites with variable chemical composition and microstructures at high-pressure and high-temperature conditions across the antigorite dehydration (1.5 GPa, 620–670 °C). For these conditions, our thermodynamic models predict the formation of olivine from magnetite decomposition and partial dehydration of antigorite. Detailed analyses of the run products reveal limited magnetite decomposition. Antigorite dehydration is restricted to samples that have been deformed. Nano-sized olivine and orthopyroxene formed locally in oblique dehydration bands and exhibit neither a clear crystallographic preferred orientation nor a topotactic relation with precursor antigorite. We argue that limited local dehydration in our experiments is related to strain and variations in reaction kinetics. Systematic investigation excludes mineralogical and chemical heterogeneities, and temperature gradients as reaction driving potentials. The structural relation of the dehydration bands suggests deformation-related dehydration, which is supported by numerical simulations that couple reaction kinetics with mechanical work rate and self-consistently predict dehydration bands. In this scenario, strain concentration due to applied axial stress locally increases the internal energy of antigorite to reach the activation energy of the dehydration reaction, enabling dehydration. This study highlights the importance of coupled mechanical and chemical processes and provides a mechanistic framework for deformation-induced dehydration of antigorite.

**Supplementary Information:**

The online version contains supplementary material available at 10.1007/s00410-025-02255-z.

## Introduction

The deep Earth water cycle involves several key processes; namely the subduction of hydrated lithosphere, the release and migration of fluids, and the resulting mantle metasomatism and island arc volcanism. Hydrous minerals are critical in this cycle as they influence the rheology of the subducting lithosphere and determine the total amount of fluids released from the slab. These fluids transport dissolved components from the slab into the mantle wedge, contributing to the deep carbon and sulfur cycles, influencing the isotopic signal and redox state of ultramafic rocks and island arc magmas, and (re-)enrich the mantle wedge with trace elements (Kodolanyi et al. 2012; Scambelluri et al. 2019; Padrón-Navarta et al. 2023; Cannaò and Debret 2024; Schwarzenbach et al. 2024; Ulrich et al. 2024). Despite its importance, the mechanisms and processes that control fluid release remain enigmatic.

Dehydration reactions, like any other mineral reactions, result from changes in external parameters that push the system out of its thermodynamic equilibrium state. Such external parameters include temperature, pressure, stress as well as external fluids (Schmidt and Poli 2014; Tajčmanová et al. 2015; Wheeler 2018; Huber et al. 2022). In this contribution, we focus on the dehydration of antigorite, a key reaction in the deep Earth water cycle. Antigorite, the high-pressure phase of the serpentine group minerals, has 13–15 wt% structurally-bound H_2_O that is released at elevated pressure and temperature (*PT*) conditions. Evidence for natural antigorite dehydration is found in various ophiolites around the world, such as the Zermatt-Saas ophiolite, Erro Tobbio unit, Nevado-Filábride complex, and Sanbagawa belt (Padrón-Navarta et al. 2011; Scambelluri et al. 2016; Fukumura et al. 2019; Gilio et al. 2019). Experimental studies have determined the thermal stability limit of antigorite as a function of pressure, temperature, composition, and oxygen fugacity (Wunder and Schreyer 1997; Bromiley and Pawley 2003; Merkulova et al. 2016; Iacovino et al. 2020; Maurice et al. 2020; Evans and Frost 2021; Eberhard et al. 2023a). Thermodynamic and mass balance calculations have been used to investigate phase relations and quantify the progressive fluid release during subduction.

Such equilibrium thermodynamic approaches, however, have limitations as they do not include reaction kinetics and non-hydrostatic conditions, and hardly account for rock heterogeneities. Natural rocks are rarely homogeneous and mineralogical, chemical and/or structural heterogeneities significantly impact the onset and progress of dehydration and fluid migration. Natural metaserpentinites show dehydration veins evidencing local scale reaction and fluid drainage (Plümper et al. 2017; Huber et al. 2022; Jabaloy-Sánchez et al. 2022; Muñoz-Montecinos et al. 2024). Experiments revealed that dehydration often starts along grain boundaries and can be related to mineralogical heterogeneities such as carbonates, sulfides, and (hydr)oxides (Eberhard et al. 2023a, b; Menzel et al. 2025). Dehydration may further be promoted by grain size reduction and in localized shear zones (Takahashi et al. 2011; French et al. 2019; Eberhard et al. 2024). Therefore, a complete description of dehydration onset and progress requires coupling of microstructures, deformation and mineral reactions in so-called hydro-mechanical-chemical (HMC) models that also account for reaction kinetics.

In this contribution we systematically investigate various driving potentials of antigorite dehydration. We performed high-pressure and high-temperature experiments on natural antigorite-serpentinites at conditions of incipient dehydration and present detailed analyzes of the chemical and mineralogical composition of the run products and the resulting microstructures. We discuss dehydration in the context of internal sample heterogeneities as well as externally induced driving potentials such as temperature gradients, which are inherent in our experiments and quantified with a numerical model, and differential stress. In combination with a numerical HMC model, our results show that the axial force applied by the piston plays a crucial role in the dehydration of our samples, and we conclude that the mechanical work resulting from differential stresses significantly enhances the release of fluids from hydrous minerals.

## Methods

### Experiments

**Fig. 1:**
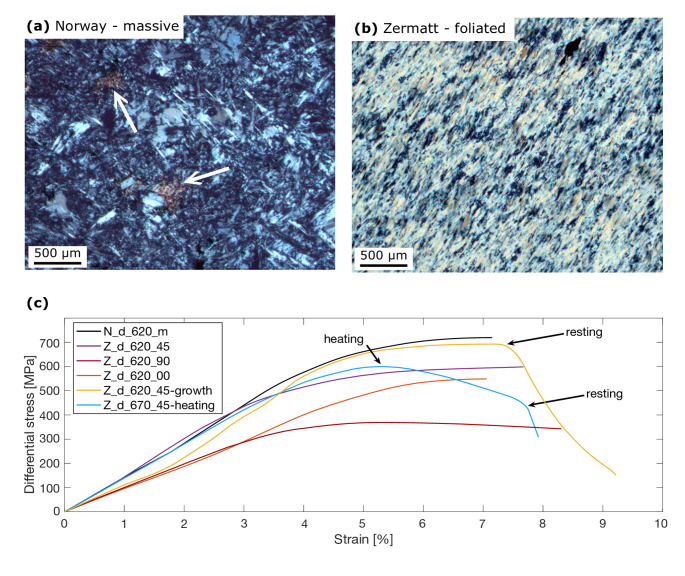
**a** Optical image of the non-foliated starting material from Norway. This starting material shows a cross-hatched pattern of antigorite flakes. Minor calcite is present in the sample (white arrow). **b** Optical image of the Zermatt starting material showing strong foliation. Full thinsection scans can be found in the data repository. **c** Stress-strain curves of coaxial compression experiments. Major stress drops are not observed in any of the experiments

We performed co-axial compression experiments at 1.5 GPa and 620–670 °C in a Tullis-modified Griggs apparatus at the Earth Sciences Institute of Orléans. Drill cores (diameter: 6.3 mm; length: ~10 mm) were prepared from two natural serpentinites that differ in their microstructure and chemical composition (cf. supplementary material *A* for detailed characterization). The serpentinite from Linnajavri, Norway, (Beinlich et al. 2012) consists of antigorite with minor magnetite and calcite. Antigorite does not have an overall crystal preferred orientation, but shows a cross-hatched pattern (Fig. 1a) interspersed with antigorite veins. Antigorite is chemically heterogeneous with variable Al, Fe and Cr content (Fig. S1). The serpentinite from Zermatt, Switzerland, contains about 2 wt% magnetite. Few grains of diopside and magnesite have been determined by optical microscopy. This sample, described in detail in Eberhard et al. (2023a, b), shows a macroscopic foliation (Fig. 1b) and exhibits a remarkable chemical homogeneity (Fig. S2). Metamorphic and/or relict olivine is not observed in either of the starting materials.

The drill cores were wrapped into a 0.025 mm thick Al- or Ni-foil to prevent Fe-alloying with the capsule and placed into welded Pt capsules. The orientation of the drill cores prepared from the Zermatt starting material was varied between 0°, 45° and 90° with respect to the macroscopic foliation. Table 1 summarizes the conditions for each experiment. We used NaCl as pressure medium, except for N_nd_650_m-KI, for which we used considerably weaker potassium iodine (Pec et al. 2012). The temperature was controlled by a type-K thermocouple. Three experiments were run under hydrostatic conditions. Unfortunately, in one of these experiments (Z_nd_650_45), high differential stresses were reached during incipient compression (> 100 MPa at 100 °C for about 2 days) due to a stuck piston, causing the sample to deform permanently. We released the stress and after checking the piston position, the experiment was run as a hydrostatic experiment (note that the sample already accumulated significant strain but was not broken). For co-axial compression experiments we applied an axial stress after equilibrating the sample at run *PT* conditions for about 30 min. The strain rate was on the order of 10^− 6^ s^− 1^ and the total finite strain was 7–9%. In Z_d_620_45-growth, we aimed to accelerate the dehydration reaction and mineral growth by fluid drainage. We added a 1 mm thick layer of diamond powder (200 μm grain size) at the bottom of the sample capsule and the sample was kept at *PT* conditions for 4 days after reaching 7.5% strain. The porous diamond layer does not compress during the experiment but allows released fluid to drain from the serpentinite drill core. In Z_d_670_45-heating, we increased the temperature from 620 °C to 670 °C during deformation to ensure that dehydration does not take place during equilibration prior to the onset of deformation. After recovery, the experimental samples were embedded in epoxy and cut and polished perpendicular to the shear plane and macroscopic foliation for microscopic analyses.

Pressure, force and displacement were continuously logged with high temporal resolution (1 Hz) during the experiment and can be found in the data repository. Force and displacement data were corrected for friction, rig stiffness and sample area, assuming homogeneous compression and a Poisson’s coefficient of 0.5. To quantify temperature gradients in our experiments arising from the cylindrical geometry of the assembly, we simulated the temperature field numerically by using the model setup by Moarefvand et al. (2021). More details on the numerical simulation are given in the supplementary material *B*.

**Table 1:** Experimental conditions

Run	Starting material	Label	α	Temperature	Confining pressure	Max. differential stress	Bulk strain rate	Total strain	Total time	reaction progress
				°C	kbar	MPa	10^− 6^ s^− 2^	%	h	
650	massive	N_nd_650_m	m	650	15	-	-	-	67 [-]	x
651	massive	N_d_620_m	m	620	15	720	1.35	7.2	41 [15]	x
653	foliated	Z_nd_650_45	45°	650	15	> 100*	-	-	24 [-]	xx
655	foliated	Z_d_620_45	45°	620	15	598	1.41	7.75	45 [15]	xx
678	foliated	Z_d_620_90	90°	620	15	370	1.46	8.3	38 [13]	xx
679	massive	N_nd_650_m-KI	m	650	15	-	-	-	63 [-]	-
680	foliated	Z_d_620_00	0°	620	15	549	1.38	7.1	35 [14]	x
681	foliated	Z_d_620_45-growth	45°	620	15	695	0.23	9.2	139 [108]	xx
682	foliated	Z_d_670_45-heating	45°	620–670	15	601	1.33	7.9	45 [16]	xx

Label refers to name of the sample as used in the text. It is composed of tags indicating the starting material as N = Norway (massive) and Z = Zermatt (foliated), axial compression (nd = no differential stress, d = differential stress), followed by the temperature, the orientation of the foliation (α), and additional information. α refers to the orientation of the macroscopic foliation with respect to the drill core axis, whereby m indicates massive serpentinite without foliation. Total time indicates the time at temperature, which includes equilibration and compression. The number in brackets is the time after reaching the hit point. Qualitative reaction progress estimated from dehydration band density is indicated as x = minor, xx = multiple through-going bands (cf. Supplementary material F). * high differential stress reached during incipient compression

### Analytical techniques

The starting materials were characterized by polarized light microscopy. Thin section scans can be found in the data repository. The experimental run products were analyzed with scanning electron microscopic techniques. We used a Zeiss Gemini 450 SEM operated at 15 kV and 1–2 nA at Utrecht University to acquire backscattered electron (BSE) maps of each sample. Phases were identified qualitatively with energy-dispersive X-ray (EDX) analyses (supplementary material *D*). The chemical composition of antigorite in the starting materials as well as in representative run products was determined by electron microprobe analyses (EMPA). We used a JEOL JXA-8530 F microprobe at Utrecht University to collect compositional maps (supplementary material *A* and *D*). Beam conditions were 20 kV and 30 nA and a spot size of 7–8 μm. The maps were quantified using XMapTools 4.3 (Lanari et al. 2019, 2024).

Occurrences of dehydration reaction in the experimental run products were localized manually in the BSE maps, whereby a certain bias introduced due to the reliance on visual inspection was accepted. The optical detection of reaction product phases in BSE maps strongly depends on the magnification, and we ensured to work strictly with the same magnification and zoom factor for consistency. The orientation of the dehydration bands (supplementary material *F*, see *Results* section for definition*)* was determined with the MATLAB toolbox FracPaQ (Healy et al. 2017). Importantly, this approach exclusively describes the position and orientation of the dehydration bands but not their width and hence does not provide quantitative information on the overall dehydration progress.

The mineralogical composition of the dehydration bands was analyzed on electron-transparent foils. Three 20 μm x 10 μm x 0.2 μm foils were prepared on representative samples (Z_d_620_90, N_d_620_m) using a FEI Helios Nanolab G3 Dual beam focused ion beam scanning electron microscope (FIB-SEM) and analyzed with Talos F200X transmission electron microscope (TEM) at Utrecht University. TEM images were acquired with 200 kV. Mineral identification was done on energy-dispersive X-ray (EDX) maps, acquired in scanning transmission electron microscope (STEM) mode. Crystal-orientation of antigorite and olivine within dehydration bands was determined on a selected area in a TEM foil prepared from sample Z_d_620_90, using automated crystal-orientation mapping technique (ACOM-TEM). Orthopyroxene was not detected in the respective map area. ACOM-TEM data were acquired with NanoMEGAS ASTAR system on a FEI Tecnai G2-20 twin at the electron microscopy facility of the Advanced Characterization Platform of the Chevreul Institute in Lille. Beam conditions were 200 kV with a nominal probe diameter of 5 nm, a step size of 10 nm x 10 nm and a precession angle of 1°. Data reduction was done with the MATLAB toolbox MTEX (Bachmann et al. 2010). Grain reconstruction with a 10° segmentation angle and subsequent removal of small grains (≤ 6 pixel) was done for olivine only, as the thickness of the foil (~ 200 nm) caused difficulties for antigorite grain reconstruction. Therefore, we calculated the kernel average misorientation (considering neighboring grains up to order 5) instead of the misorientation to the grains mean orientation. Misorientation between olivine and antigorite grains was calculated on neighboring pixels (correlated) as well as on randomly sampled pixels (uncorrelated). Orientation distribution functions were calculated for olivine only.

### Thermodynamic models

We calculated the expected phase relations in an *f*(O_2_) buffered system at FMQ (fayalite-magnetite-quartz equilibrium) with Perple_X (Connolly 1995), version 6.9.1, using the database from Holland and Powell (2011), version DS622, the modified Tait equation of state for solids and the equation of state by Pitzer and Sterner (1994) for H_2_O. We used the bulk composition representative for the Zermatt starting material, derived by Eberhard et al. (2023a): MgO = 37.2 wt%, Al_2_O_3_ = 2.4 wt%, SiO_2_ = 40.3 wt%, FeO = 6.1 wt%, H_2_O = 14 wt%. The O_2_ component is unconstrained, allowing to mimic variations in bulk redox state. Considered solid solution models are as followed: olivine - O(JH), orthopyroxene - Opx(JH) and garnet - Grt(JH) from Jennings and Holland (2015); spinel – Sp(HGP) from Holland et al. (2018); orthoamphibole - oAmph(DP) from Diener et al. (2007); chlorite - Chl(W) from White et al. (2014); antigorite - Atg(LE) from Eberhard et al. (2023a); and talc - T as ideal solid solution. An additional binary phase diagram was calculated for the same system but variable bulk Al_2_O_3_ content ranging from 0 wt% to 2.4 wt% at a fixed pressure of 1.5 GPa.

### Numerical hydro-mechanical-chemical model

We elaborate the 2D numerical HMC model presented in Schmalholz et al. (2023) that simulates the coupling of serpentinite dehydration, fluid flow, and rock deformation. The model couples results from thermodynamic equilibrium calculations, based on Gibbs energy minimization, with a two-phase transport code. A particular feature of the HMC model is that it considers the large spatial and temporal variations in solid density associated with antigorite dehydration and olivine formation (see e.g., Schmalholz et al. 2023). Here, we used a simple 3-component system (MgO - SiO_2_ - H_2_O), for which the thermodynamic equilibrium state is obtained with Perple_X (Connolly 1995). Dehydration of antigorite follows the simplified reaction:

1 $$\mathop {{\text{Mg}}_{{{\text{48}}}} {\text{Si}}_{{{\text{34}}}} {\text{O}}_{{{\text{85}}}} \left( {{\text{OH}}} \right)_{{{\text{62}}}} }\limits_{{antigorite}} \to \mathop {{\text{14 Mg}}_{{\text{2}}} {\text{SiO}}_{{\text{4}}} }\limits_{{olivine}} + \mathop {{\text{10 Mg}}_{{\text{2}}} {\text{Si}}_{{\text{2}}} {\text{O}}_{{\text{6}}} }\limits_{{orthopyroxene}} + {\text{ }}\mathop {{\text{31}}{\text{ H}}_{{\text{2}}} {\text{O}}}\limits_{fluid} . $$

The principal coupling quantity is the fluid pressure: its magnitude controls the pressure of thermodynamic equilibrium and its spatial gradients governs fluid flow according to Darcy’s law. The deviatoric shear deformation of the solid matrix is modeled as a visco-elasto-plastic solid using a pressure-insensitive Von Mises plastic yield stress. The volumetric deformation is poro-viscous. The permeability is a power-law function of the porosity, as in a Kozeny-Carman relation. The shear and compaction viscosities are porosity dependent. We consider an isothermal system, i.e., no thermal gradient exists, with a fixed chemical composition.

At the beginning of the simulation, the total pressure and fluid pressure are homogeneous and the thermodynamic pressure lies within the forsterite + enstatite stability field. The initial mineral assemblage, and hence the solid density, is composed entirely of metastable antigorite. A pure shear deformation with vertical $\:{\sigma\:}_{1}$ is applied to mimic the conditions of the co-axial compression experiments. For simplicity, we place a single initial perturbation in porosity in the model center, which causes a change of viscosity, and hence stress inhomogeneities. The variation of fluid and solid densities during the deformation is calculated using pre-calculated Perple_X results that provide the solid and fluid densities as well as the mass fraction of structurally-bound H_2_O in the minerals as a function of the fluid pressure. We use the values of the mechanical work rate, $\:W$, to calculate a kinetic time, $\:{t}_{kin}$, which we define as:

2 $$ \:t_{{kin}} = {{t_{{kin0}} } \mathord{\left/ {\vphantom {{t_{{kin0}} } {\left( {\left( {2 + tanh\left( {10\left( {\mathop W\limits^{ - } - \Delta W/5} \right)/\Delta W} \right)} \right)^{{4.5}} } \right)}}} \right. \kern-\nulldelimiterspace} {\left( {\left( {2 + tanh\left( {10\left( {\mathop W\limits^{ - } - \Delta W/5} \right)/\Delta W} \right)} \right)^{{4.5}} } \right)}}, $$

where $\:{t}_{kin0}$ is 0.00025 times the hydraulic diffusion time, $\:\stackrel{-}{W}=W-{W}_{av}$, with $\:{W}_{av}$ being the average value of $\:W$ in the model domain and $\:\varDelta\:W={W}_{max}-{W}_{min}$ with $\:{W}_{max}$ and $\:{W}_{min}$ being the maximum and minimum value of $\:W$ in the model domain, respectively. We calculated $\:W$ at every point in the 2D model domain as the product of the local square root of the second invariant of the stress tensor times the local square root of the second invariant of the strain rate tensor, $\:W={\tau\:}_{II}{\dot{\epsilon\:}}_{II}$. The $\:{t}_{kin}$ controls the temporal change of the solid density towards the thermodynamic equilibrium value in a first-order linear kinetic model (for details see Schmalholz et al. 2023). The applied formulation generates larger values of $\:{t}_{kin}$, hence slower kinetics, for smaller values of $\:W$ and smaller values of $\:{t}_{kin}$, hence faster kinetics, for larger values of $\:W$. During progressive deformation, we monitor in the numerical simulation the fluid pressure, $\:{p}_{f}$, the square root of the second invariant of the deviatoric stress tensor, $\:{\tau\:}_{II}$, the mechanical work rate, $\:W$, and the solid density, $\:{\rho\:}_{s}.$

## Results

### Experimental observations

All recovered samples, except N_nd_650_m-KI, show macroscopic ductile deformation, i.e., bulging or barreling of the capsule (Fig. S3) in agreement with the absence of larger (> 20 MPa) stress drops in the stress-strain curves (Fig. 1c). The strength of the samples does not show a systematic relation to the type of starting material. But we find that samples having the macroscopic foliation parallel (0°) and perpendicular (90°) to the maximum shortening axis do show slightly lower strength with respect to samples with 45° foliation as well as to non-foliated samples. We observe localization of deformation in all samples subjected to a differential stress. Additionally, also Z_nd_650_45, which experienced high differential stresses during incipient compression (cf. *Methods* section), shows strong localization. The alumina pistons cracked and locally punctured few of the capsules.

**Fig. 2:**
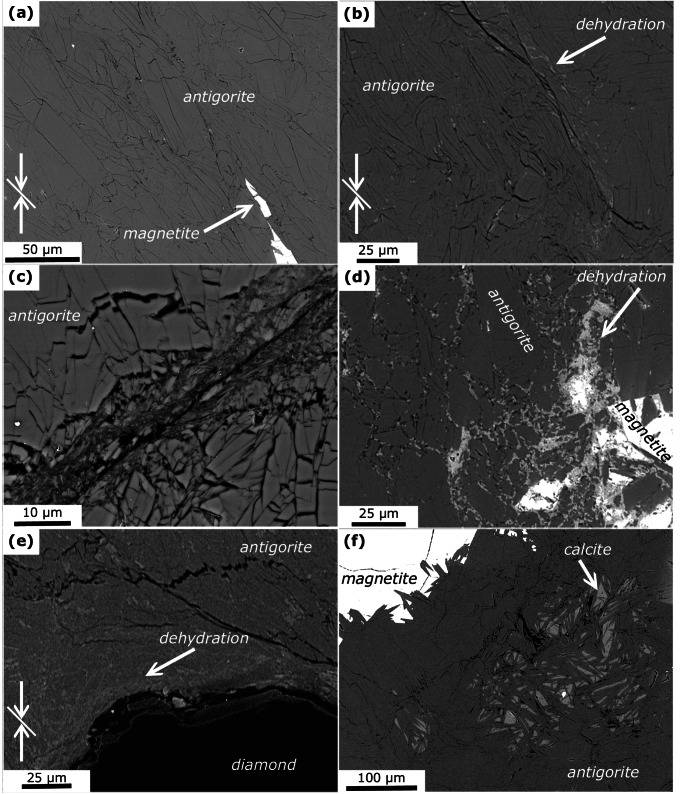
BSE images showing microstructures in run products. White arrows indicate shortening direction of non-hydrostatic experiments and white lines indicate the macroscopic foliation of Zermatt samples. **a** Z_d_620_45 shows bladed antigorite grains that exhibit a shape preferred orientation defining the macroscopic foliation. **b** Kinking and bending in Z_d_620_45. Anhydrous phases formed in the vicinity of highly deformed antigorite grains. **c** N_nd_650_m-KI showing a micro-gouge. No anhydrous phases are observed. **d** Magnetite grains in N_nd_650_m reacted locally with antigorite to form olivine. **e** Around diamond grains in Z_d_620_45-growth anhydrous phases formed. **f** Magnetite and carbonates are both not affected by dehydration in N_nd_650_m-KI

**Fig. 3:**
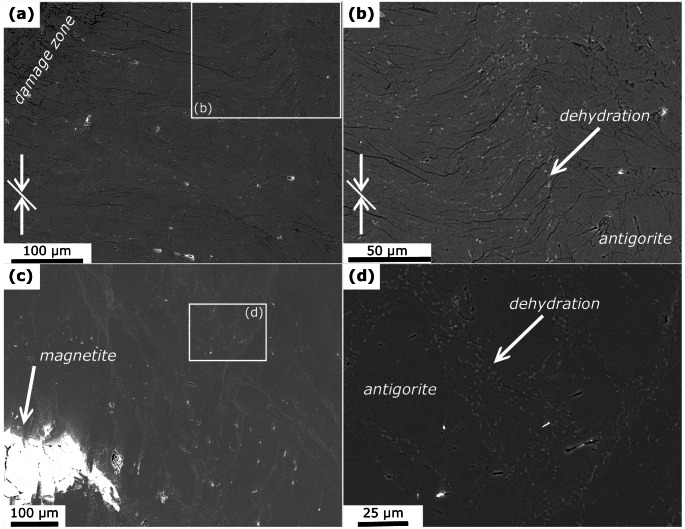
BSE images showing microstructures in run products. White arrows indicate shortening direction of non-hydrostatic experiments and white lines indicate the macroscopic foliation of Zermatt samples. **a** Anhydrous phases forming close to a damage zone in the corner of Z_d_620_45. Rectangle indicates location of **b**, showing anhydrous phases that formed locally in a kink band, while the neighboring domain is not affected by dehydration. **c** Anhydrous phases form a network of cross-cutting veins in N_nd_650_m, associated with magnetite grains. Rectangle shows location of **d**

**Fig. 4:**
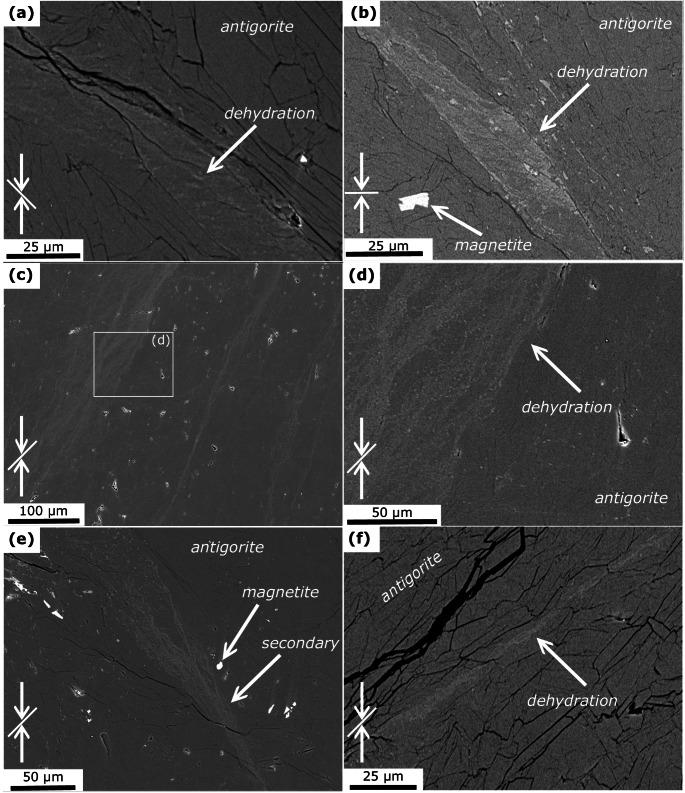
BSE images showing microstructures in run products. White arrows indicate shortening direction of non-hydrostatic experiments and white lines indicate the macroscopic foliation of Zermatt samples. **a** In Z_d_620_45 anhydrous phases formed adjacent to a fault. **b** Shortening and extension structure in Z_d_620_90. **c** Several (sub-)parallel bands of anhydrous phases in a 30° angle with respect to the compression axis in Z_nd_650_45. The indicated shortening direction belongs to deformation experienced prior to the actual experiment (cf. *Methods *section). Rectangle indicates location in **d**. **d** Typically, these bands are composed of nano-sized anhydrous phases that are clearly separated from non-dehydrated domains and are not related to fractures. **e** Diffuse anhydrous phases transition into sharp dehydration band in Z_d_670_45-heating. **f** Bands of anhydrous phases do not follow grain boundaries in Z_d_670_45-heating

Microchemical and -structural analyses reveal antigorite as the major phase in all samples (Figs. 2, 3 and 4), independent of the starting material, total strain, and run temperature. Bending and kinking of antigorite grains is frequently observed (Fig. 2b). Few samples show brittle damage zones in the corners and N_nd_650_m-KI exhibits a micro-gouge (Fig. 2c). Evidence for dehydration, i.e., formation of anhydrous phases is not observed in this sample. All other samples show anhydrous phases in various structural relations. These can be divided into 3 types, being (I) related to minor phases; (II) dispersed grains in antigorite matrix in restricted domains; and (III) sharp dehydration bands:

I) Magnetite is present as a minor phase in all recovered samples. The formation of fine-grained olivine coronas, identified from EDX analyses, around magnetite grains (Fig. 2d) is observed only in few samples. Importantly, only magnetite grains close to the capsule wall and/or associated with through-going faults and high fracture densities show olivine formation, whereas other magnetite grains within the same samples are not related to olivine. Z_d_620_45-growth shows the formation of anhydrous phases, mainly olivine, near the contact to diamond grains (Fig. 2e). On the other hand, minor calcite that is present in the non-foliated starting material from Norway shows no evidence for reaction with antigorite (Fig. 2f), except for minute formation of diopside in N_nd_650_m (supplementary material D).
II) Dispersed anhydrous phases, mainly olivine, formed in restricted domains that often indicate high strain by bent antigorite grains and are associated with brittle damage zones and kink bands (Fig. 3). In some samples, these domains reveal a transition into sharp bands described below. In N_nd_650_m anhydrous phases form a network of cross-cutting bands with an approximately 30° relation with respect to the maximum shortening axis (Fig. 3e-f, supplementary material *F*), closely resembling microstructures modelled by Baïsset et al. (2024).
III) In all samples, except N_nd_650_m-KI, anhydrous phases with grain sizes of few hundred nanometer form sharp oblique bands. These bands, to which we refer to as dehydration bands in the following, are approximately straight (Fig. 4, supplementary material *F*). Some of the dehydration bands are related to through-going faults, but typically only form on one side of the fault or are bound on two sides by a fault and do not cover the full length of the fault plane (Fig. 4a, b). Other dehydration bands are associated with kink bands. Most dehydration bands, however, show no relation to fractures and faults (Fig. 4c–f).

**Fig. 5:**
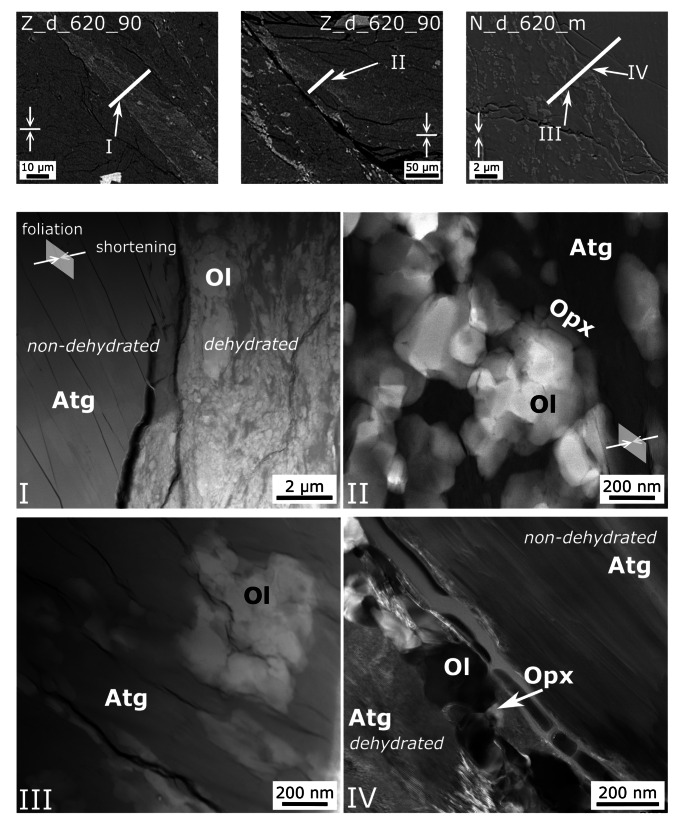
TEM analyses of representative samples. Top: BSE images showing location of FIB cuts. White arrows indicate shortening direction and white lines indicate the macroscopic foliation of Zermatt samples. Roman numbers mark the position of detailed images. (I) High-angle annular dark-field (HAADF) image showing large antigorite grains in the non-dehydrated domain and a sharp boundary to the dehydration band. (II) HAADF image showing olivine and orthopyroxene grains within a dehydration band. (III) HAADF image showing abundant microcracks in a dehydration band. (IV) Dark-field image of antigorite in a dehydration band with strong deformation in contrast to antigorite outside of the band. Mineral abbreviation as followed: Atg - antigorite, Ol - olivine, Opx - orthopyroxene

**Fig. 6:**
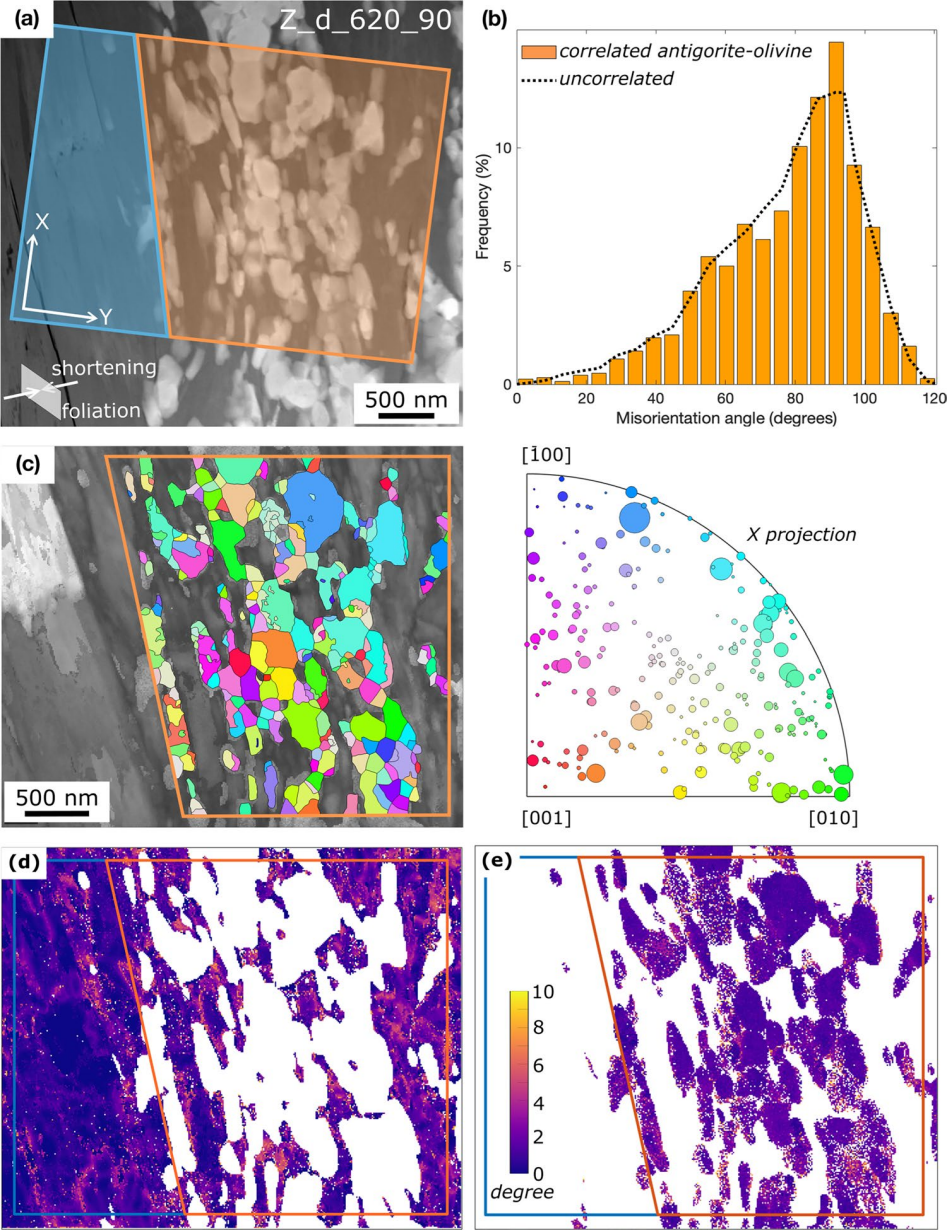
ACOM-TEM results. **a** HAADF image showing location of the orientation map in relation to the shortening direction. The orientation map was split into a non-dehydrated and a partially dehydrated domain. **b** Frequency plot of misorientation between correlated (neighboring) antigorite-olivine pairs and uncorrelated (random) pixels. No special orientations are observed. **c** IPF colors (X-projection) showing mean orientation of reconstructed olivine grains (point group Pbnm) **d** Kernel average misorientation of antigorite. **e** Kernel average misorientation of olivine

Due to the small grain size of the anhydrous reaction products, the mineralogy was determined by TEM analyses. (Fig. 5). The contact between the dehydration bands and the non-reacted domains is typically sharp. The dehydration bands are composed of nanometer-sized olivine + orthopyroxene with relict antigorite, showing strong grain size reduction. The porosity in these bands is low, while microcracks are abundant. ACOM-TEM data Fig. 6a–c, supplementary material *E*) reveal only very weak preferred orientation of olivine with the b axis perpendicular to the shortening direction. A clear topotactic relation between olivine and antigorite is not observed, as shown by similar frequency distribution of correlated (neighboring) and uncorrelated (random) antigorite-olivine pixels. The computed kernel average misorientation indicates low grain internal deformation for olivine. Antigorite overall does not reveal significantly higher strain within the dehydration band compared to the larger grains in the non-reacted domain (Fig. 6d–e). The latter, however, should be treated with caution due to the small field of view and the limited number of grains measured outside of the dehydration bands.

**Fig. 7:**
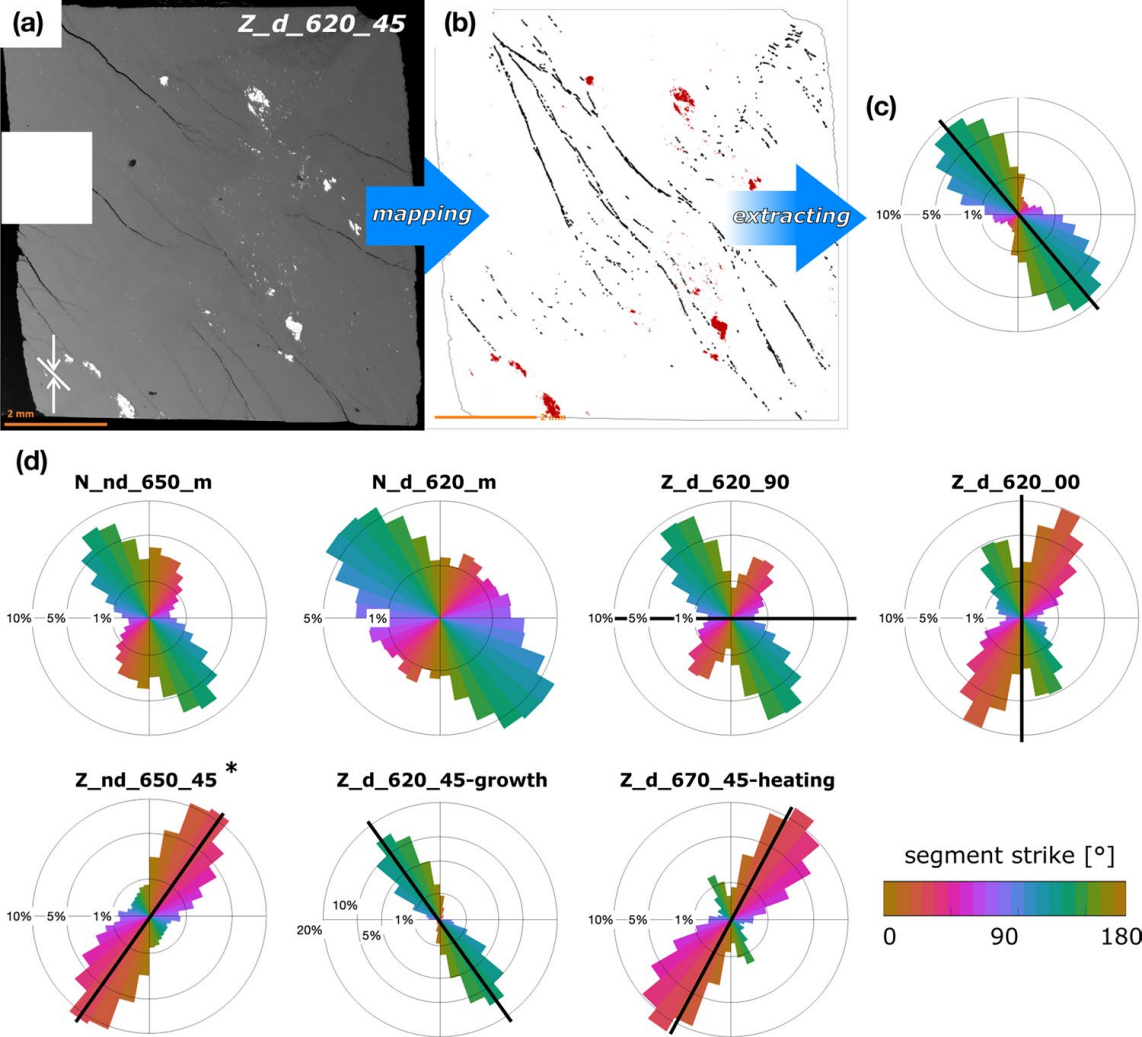
Workflow of dehydration band orientation mapping. **a** BSE images were used to locate dehydration. White arrows indicate shortening direction and white line indicates the macroscopic foliation. **b** Black lines represent dehydration bands and red spots are magnetite grains used for reference. **c** Rose plot showing the orientation of dehydration bands. Black line shows the macroscopic foliation. **d** Compilation of vein orientation in all samples, except N_nd_650_m-KI that did not exhibit any dehydration. * Z_nd_650_45 was deformed during incipient compression (cf. *Methods* section). A detailed representation of the veins in each sample can be found in the supplementary material *D*

The orientation of dehydration bands with respect to the maximum shortening axis is summarized in Fig. 7 and shows a consistent angle of 30–45°, independent on the starting material and temperature of the experiment. In samples with 45° foliation, the orientation of the dehydration bands matches the foliation. Samples with 0° and 90° foliation show more pronounced conjugate bands. Rotation of the bands due to kink fold formation is observed in foliated samples, most notably in Z_d_620_90 (Fig. S9).

### Thermal gradient calculation

**Fig. 8:**
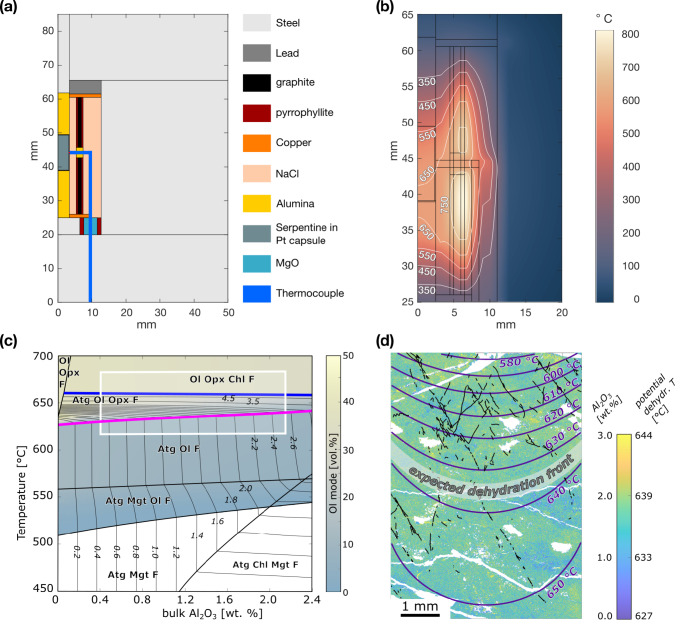
**a** Setup of the numerical temperature calculation showing the geometry and materials of the sample assembly. Note that only half of the assembly was computed. **b** The numerical simulation shows an absolute temperature gradient of ~ 100 °C between the top and bottom of the sample. **c** Binary phase diagram at 1.5 GPa calculated for antigorite bulk composition with variable bulk Al_2_O_3_ content ranging from 0 wt% to 2.4 wt%. Mineral abbreviation as followed: Atg - antigorite, Chl - chlorite, Ol - olivine, Opx - orthopyroxene, Mgt - magnetite, F - fluid. Color coding indicates the mode of olivine, serving as a proxy for antigorite dehydration. The contours indicate Al_2_O_3_ [wt%] of antigorite. The rectangle highlights the thermal and compositional range covered in our experiments. The thick pink line shows the onset of antigorite dehydration by reaction 1, the thick blue line indicates the final antigorite-out. **d** Antigorite Al content in the center part of N_nd_650_m measured by EPMA converted into a potential dehydration temperature map. Based on the experimental temperature gradient (purple isogrades), dehydration by reaction 1 is expected only in the lower part of the sample and does not correlate with observed distribution of dehydration bands (black lines)

During the experiment all samples experienced a temperature gradient. The numerical model shows that the temperature field in experiments run at 650 °C (thermocouple temperature) has an hourglass shape. The maximum temperature difference in the capsule is about ~ 100 °C with the hot spot slightly below the thermocouple (Fig. 8a, b). The potential dehydration temperature, i.e., the temperature at which the first olivine from reaction 1 is formed, varies by less than 20 °C between low- and high-Al antigorite used in this study (Fig. 8c). Together, these findings predict a relatively sharp dehydration front perpendicular to the cylinder axis (Fig. 8d) and partial dehydration in the lower part of samples that were heated to 650 °C (thermocouple temperature). Samples that were heated to 620 °C (thermocouple temperature) are expected to remain below the dehydration temperature and the sample heated to 670 °C (thermocouple temperature) is expected to show partial dehydration over the full sample length.

#### Numerical hydro-mechanical-chemical modelling

**Fig. 9:**
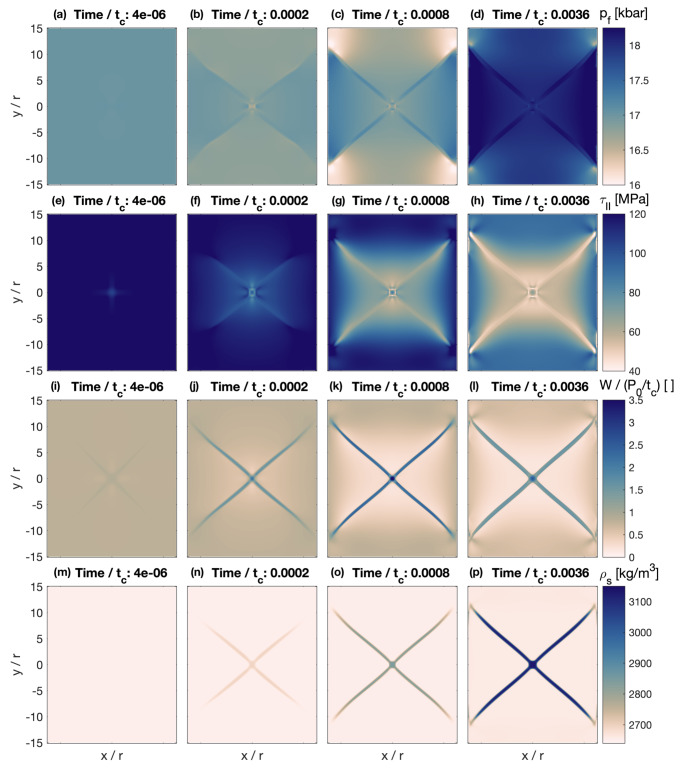
Numerical results of HMC model. Panels **a**–**d** show the evolution of the fluid pressure $\:{p}_{f}$ at four different time steps; **e**–**h** show the evolution of the square root of the second invariant of the deviatoric stress tensor $\:{\tau\:}_{II}$; **i**–**l** show the evolution of the mechanical work rate $\:W$; and **m**–**p** show the evolution of the solid density $\:{\rho\:}_{s}$

Figure 9 shows 4 different time steps (indicated in dimensionless time) of the HMC model during progressive deformation. After the first time step, there are hardly visible variations in $\:{p}_{f}$ in the model center due to the viscosity perturbation (Fig. 9a). Values of $\:{\tau\:}_{II}$ also vary around the center but are homogeneous in most of the model domain (Fig. 9e). There are four shear bands slightly visible in the distribution of $\:W$ that is higher inside the bands than outside (Fig. 9i). These shear bands have angles of ~ 45° with respect to the vertical direction of $\:{\sigma\:}_{1}$. No variations are yet visible in $\:{\rho\:}_{s}$, having the value corresponding to antigorite (Fig. 9m). With progressive time and deformation, $\:{p}_{f}$ in the evolving shear bands is slightly larger than outside (Fig. 9a–d). Outside the shear bands, $\:{p}_{f}$ is first slightly decreasing and then increasing due to the fluid release during the dehydration that generates a slight fluid overpressure in the model domain. In this model the shear and compaction viscosities are porosity dependent with larger porosities causing smaller viscosities. The reaction-induced porosity increase in the shear bands causes an effective viscosity weakening that further localizes the deformation. Around the top and bottom regions of the model, $\:{\tau\:}_{II}$ remains approximately constant with time and values of $\:{\tau\:}_{II}$ are slightly smaller inside the shear bands than outside (Fig. 9e–h). Inside the evolving shear bands, $\:W$ is always larger than outside (Fig. 9i–l). During the simulation, the values of $\:{\rho\:}_{s}$ increase from ~ 2650 (density of antigorite) to ~ 3150 kg/m^3^ (density of olivine + orthopyroxene) inside the shear bands (Fig. 9m–p) because the kinetic time is controlled by $\:W$ (see Eq. 1) and larger values of $\:W$ cause a faster reaction (i.e., shorter kinetic time). The shear bands, hence, mimic the diffuse dehydration bands observed in the axial compression experiments.

## Discussion

**Fig. 10:**
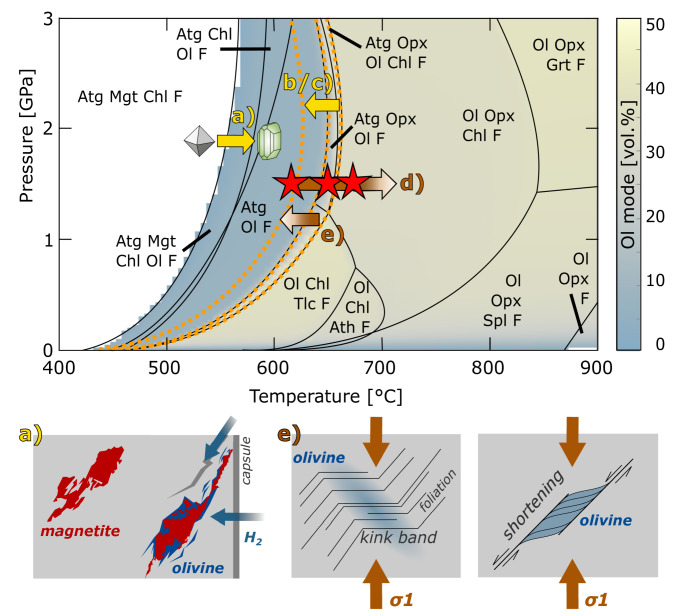
Pseudosection of a serpentinite buffered at FMQ. The color code indicates vol% of olivine, forming upon continuous serpentine and chlorite dehydration. Mineral abbreviation as followed: Atg - antigorite, Chl - chlorite, Ol - olivine, Opx - orthopyroxene, Mgt - magnetite, Spl - spinel, Grt - garnet, Tlc - talc, Ath - anthophyllite, F - fluid. Red stars indicate the experimental conditions. Labeled arrows highlight the different driving potentials for dehydration (yellow: internal sample heterogeneities; brown: externally induced) discussed in this study: **a** the presence of minor phases, **b** crystallographic variations, **c** chemical composition, **d** temperature, and **e** mechanical driving potentials. See text for a detailed discussion. Bottom row sketches the observed olivine in our experiments. Magnetite-related olivine (cf. Figure 2d) forms in regions with elevated H_2_ flux close to capsule walls and fractures, strain-related olivine (cf. Figures 3 and 4) forms e.g. in kink bands and combined shortening and extension structures

The pseudosection in Fig. 10 shows the equilibrium phase relations for our Zermatt bulk composition. At 1.5 GPa the lowest temperature occurrence of olivine in an open system is marked by a magnetite-out reaction at 520 °C to 550 °C and we would expect olivine to form locally from magnetite decomposition in all experiments. Olivine formation according to reaction 1 is expected to occur only at temperatures ≥ 635 °C, restricting reaction 1 to samples equilibrated at and above 650 °C (thermocouple temperature).

Except for N_nd_650_m-KI, all our samples show the formation of antigorite-dehydration products, but their spatial distribution is not uniform and does not match the expected phase relations: we observe olivine + orthopyroxene forming from reaction 1 in restricted domains and bands. Even if an uncertainty of ± 20 °C for the thermocouple is assumed, a clear relation between reaction progress and experimental temperature (Table 1) cannot be observed. For example, Z_d_670_45-heating was deformed at temperature of 670 °C but shows similar reaction progress to samples Z_d_620_45, Z_d_620_90 and Z_d_620_45-growth equilibrated at lower temperatures (Table 1, supplementary material *F*). On the contrary, despite the experimental temperatures exceeding the calculated magnetite-out reaction (Fig. 10), magnetite-related olivine (Fig. 2) is only observed in few samples and typically does not affect all magnetite grains within a single sample.

We suggest that the distinct distribution of the observed dehydration products in our experiments, specifically the formation of dehydration bands, is to a great extent controlled by the reaction kinetics and the applied differential stress, which are not considered in thermodynamic equilibrium calculations. The reaction kinetics can be related, based on the transition state theory, to the difference in the Gibbs free energy of activation $\:{\Delta\:}{G}^{\ddagger}$, the so-called energy barrier, between the transition state and the reactants (Lasaga 1998):

3 $$\:k\left(T\right)=\kappa\:\frac{{k}_{B}T}{h}{exp}^{\frac{-{\Delta\:}{G}^{\ddagger}}{RT},}$$

where *T* is the temperature, *k(T)* the temperature-dependent rate constant, *κ* the transmission coefficient, *k*_*B*_ the Boltzmann constant, *h* the Plank constant and *R* the universal gas constant. At constant temperature and hydrostatic pressure, the standard $\:{\Delta\:}{G}^{\ddagger}$ can be expressed as:

4 $$\:\varDelta\:{G}^{\ddagger}=\:\varDelta\:{H}^{\ddagger}-T\varDelta\:{S}^{\ddagger},$$

where $\:\varDelta\:{H}^{\ddagger}$ is the activation enthalpy and $\:\varDelta\:{S}^{\ddagger}$ the activation entropy. The potential energy landscape of the system that controls the energy barrier of a reaction can be altered due to local chemical variations of the system and the presence of defects in crystals. Additionally, non-hydrostatic stresses may affect the energy of solids (Gibbs 1876). The magnitude of the displacements of reactions under stress and the magnitude of the effect of stress on kinetics, are still debated (Frolov and Mishin 2010; Mazzucchelli et al. 2024; Wheeler 2020) and to our knowledge, a complete kinetic theory for a system under anisotropic stress is still lacking.

In the following, we systematically discuss how the intrinsic mineralogical, crystallographic and chemical heterogeneities of our samples correlate with the observed microstructures and could explain the localized dehydration reaction. We further qualitatively discuss the possible effects of external factors on the reaction kinetics, being the thermal gradient and the applied differential stress.

### Dehydration due to internal sample heterogeneities

#### Mineralogical heterogeneities

Minor phases such as magnetite, brucite and calcite, which may be present in serpentinites, can react with the antigorite upon subduction and release fluid (mechanism *a* in Fig. 10). The attribute ‘minor‘ implies that these phases are not rock-forming and fluid release occurs only locally. Such local dehydration reactions can impose stress on the surrounding grains (Jung et al. 2004; Ferrand et al. 2017; Schmalholz et al. 2020) and may lead to microcracks that further evolve into bands and veins.

With few exceptions magnetite is systematically present in serpentinites (Frost and Beard 2007; Klein et al. 2014; Evans et al. 2017) and can react with antigorite to form olivine according to the reaction.

5 $$\begin{gathered} \mathop {{\text{Mg}}_{{{\text{48}}}} {\text{Si}}_{{{\text{34}}}} {\text{O}}_{{{\text{85}}}} \left( {{\text{OH}}} \right)_{{{\text{62}}}} }\limits_{{antigorite}} + \mathop {{\text{6.66}}{\text{ Fe}}_{{\text{3}}} {\text{O}}_{{\text{4}}} }\limits_{{magnetite}} + \mathop {{\text{ 6.66}}{\text{ H}}_{{\text{2}}} } \hfill \\ \to \mathop {{\text{34 }}\left( {{\text{Mg}},{\text{Fe}}} \right)_{{\text{2}}} {\text{SiO}}_{{\text{4}}} }\limits_{{olivine}} + {\text{ }}\mathop {{\text{37.66}}{\text{ H}}_{{\text{2}}} {\text{O}}}\limits_{{fluid}} \hfill \\ \end{gathered} $$

This reaction requires infiltration of external H_2_ and is observed in various serpentine experiments (Merkulova et al. 2016; Menzel et al. 2025). Accordingly, in our experimental setup, any moisture present in the compression medium reacts with the graphite furnace to form H_2_ via the reactions C + H_2_O = CO + H_2_ and C + 2 H_2_O = CO_2_ + 2 H_2_. The resulting gradient in chemical potential of H_2_ drives hydrogen diffusion through the platinum capsule into the sample. Metamorphic olivine related to magnetite formed only in few samples close to fractures in the sample and along the capsule wall, regions with a higher hydrogen flux, but complete consumption of magnetite grains is not observed. This suggest that the kinetics of magnetite decomposition according to reaction 6 is relatively high and H_2_ diffusion is the rate limiting process. Reaction 5 can therefore explain the formation of magnetite-related olivine but not dehydration in restricted domains and bands.

Several studies propose that brucite, formed during serpentinization of ultramafic rocks (Debret et al. 2019; McCollom et al. 2020a, b) or during lizardite-antigorite transition (Menzel 2018), reacts with antigorite and leads to the formation of metamorphic olivine veins (Plümper et al. 2017; Huber et al. 2022; Menzel et al. 2025) according to the simplified reaction.

6 $$ \begin{gathered} \mathop {{\text{Mg}}_{{{\text{48}}}} {\text{Si}}_{{{\text{34}}}} {\text{O}}_{{{\text{85}}}} \left( {{\text{OH}}} \right)_{{{\text{62}}}} }\limits_{{antigorite}} + {\text{ }}\mathop {{\text{2}}0{\text{ Mg}}\left( {{\text{OH}}} \right)_{{\text{2}}} }\limits_{{brucite}} \hfill \\ \to \mathop {{\text{34 Mg}}_{{\text{2}}} {\text{SiO}}_{{\text{4}}} }\limits_{{olivine}} + {\text{ }}\mathop {{\text{51 H}}_{{\text{2}}} {\text{O}}{\text{.}}}\limits_{{fluid}} \hfill \\ \end{gathered} $$

At typical subduction *PT* gradients, reaction 6 takes place at ~ 500 °C. The non-foliated serpentinite used in this study likely did not experience such high temperatures. Although we did not observe brucite in the starting material, we cannot exclude brucite intergrown with antigorite on a nanometer scale (Malvoisin et al. 2021), which may result in dispersed olivine formation. Nonetheless, dispersed olivine formation is also observed in samples with Zermatt starting material (Fig. 3a, b). Peak *PT* conditions experienced by serpentinites from the Zermatt region were far above the brucite-out reaction (Li et al. 2004; cf. supplementary material *A*), which indicates that reaction 6 cannot be responsible for the observed dispersed olivine formation and likewise for the dehydration bands.

Finally, calcite-antigorite reactions should occur at our experimental conditions and diopside + olivine formation along with antigorite consumption is expected (Menzel et al. 2019; Eberhard et al. 2023b). The reaction between antigorite and calcite, taking place at temperatures < 500 °C at 1.5 GPa, can be written in the simplified form.

7 $$ \begin{gathered} \mathop {{\text{Mg}}_{{{\text{48}}}} {\text{Si}}_{{{\text{34}}}} {\text{O}}_{{{\text{85}}}} \left( {{\text{OH}}} \right)_{{{\text{62}}}} }\limits_{{antigorite}} + {\text{ }}\mathop {{\text{2}}0{\text{ CaCO}}_{{\text{3}}} }\limits_{{calcite}} \to \mathop {{\text{1}}0{\text{ MgCaSi}}_{{\text{2}}} {\text{O}}_{{\text{6}}} }\limits_{{diopside}} ~ \hfill \\ + {\text{ }}\mathop {{\text{14 Mg}}_{{\text{2}}} {\text{SiO}}_{{\text{4}}} }\limits_{{olivine}} + {\text{ }}\mathop {{\text{1}}0{\text{ CaMg}}\left( {{\text{CO}}_{{\text{3}}} } \right)_{{\text{2}}} }\limits_{{dolomite}} + {\text{ }}\mathop {{\text{31 H}}_{{\text{2}}} {\text{O}}}\limits_{{fluid}} \hfill \\ \end{gathered} $$

Calcite is observed in all non-foliated samples. However, diopside formation occurred only in N_nd_650, while carbonates in N_d_620_m and N_nd_650_m-KI are not affected by reaction 7, indicating generally slow reaction kinetics for this reaction.

#### Crystallographic heterogeneities

Several studies suggest that pressure and temperature can influence the polysome length of antigorite (Mellini et al. 1987; Shen et al. 2020). It is typically assumed that polysomes with lower *m*-values are stable at higher temperatures and we can write the reaction

8 $$ \begin{gathered} \mathop {{\text{Mg}}_{{{\text{3m}} - {\text{3}}}} {\text{Si}}_{{{\text{2m}}}} {\text{O}}_{{{\text{5m}}}} \left( {{\text{OH}}} \right)_{{{\text{4m}} - {\text{6}}}} }\limits_{{antigorite{\text{ }}m}} \to \hfill \\ \mathop {{\text{Mg}}_{{{\text{3}}({\text{m}} - {\text{x}}) - {\text{3}}}} {\text{Si}}_{{{\text{2}}({\text{m}} - {\text{x}})}} {\text{O}}_{{{\text{5}}({\text{m}} - {\text{x}})}} \left( {{\text{OH}}} \right)_{{{\text{4}}({\text{m}} - {\text{x}}) - {\text{6}}}} }\limits_{{antigorite{\text{ }}m - x}} \hfill \\ + {\text{ }}\mathop {{\text{x Mg}}_{{\text{2}}} {\text{SiO}}_{{\text{4}}} }\limits_{{olivine}} + \mathop {{\text{x}}/{\text{2 Mg}}_{{\text{2}}} {\text{Si}}_{{\text{2}}} {\text{O}}_{{\text{6}}} }\limits_{{pyroxene}} + \mathop {{\text{2x H}}_{{\text{2}}} {\text{O}}}\limits_{{fluid}} , \hfill \\ \end{gathered} $$

where *m* corresponds to half of the tetrahedral positions along the crystallographic *a* axis, and *x* is an integer modulating the polysome length. The exact relation between the *m*-value, temperature and reaction kinetics is unknown and a thermodynamic description of reaction 8 at our experimental conditions is still lacking (Ritterbex and Plümper 2024; Tsuchiya et al. 2024). If variations in the polysome length occurred in the starting materials and led to partial dehydration according to reaction 8 (mechanism *b* in Fig. 1), we expect to see recrystallisation of antigorite to lower *m*-polysomes accompanying olivine formation. Figure 5 indicates strong grain size reduction of antigorite in dehydration bands, but we do not see evidence for recrystallisation. Therefore, it is unlikely that reaction 8 caused the formation of olivine and orthopyroxene in our samples.

#### Chemical heterogeneities

The dehydration temperature of antigorite depends on its chemical composition (Bromiley and Pawley 2003; Merkulova et al. 2016). We discuss here whether local chemical changes in antigorite composition could cause local dehydration (mechanism *c* in Fig. 10). Chemical analyses of the antigorite in the non-foliated starting material from Norway revealed substantial variability in Al and Cr content, but not in Fe (Fig. S1). The effect of Cr on antigorite stability is so far unknown and to our knowledge no thermodynamic data on a Cr-endmember exist. Therefore, we focus here on the Al content alone. Thermodynamic calculations show that the Al content in serpentinites is a function of temperature if element exchange with another Al-bearing phase, such as chlorite, occurs (Padrón-Navarta et al. 2013; Eberhard et al. 2023a). Since our starting materials do not contain chlorite, the chemical composition of antigorite with respect to Al is fixed and we can use the antigorite Al content to estimate the local potential dehydration temperature. N_nd_650_m shows patches with a higher Al content (> 2 wt% Al_2_O_3_) that can be distinguished from Al-poor (< 0.5 wt% Al_2_O_3_) patches. Based on thermodynamic relations derived from Fig. 8c, we convert the antigorite Al content into a temperature map (Fig. 8d), outlining the minimum temperature for olivine formation from reaction 1. High-Al patches are expected to dehydrate at ~ 640 °C, whereas low Al-patches already dehydrate at 625 °C. In Fig. 8d we compare the antigorite Al content with the temperature gradient and the distribution of dehydration bands in sample N_nd_650_m, but do not find a correlation. Furthermore, the Zermatt starting material has a remarkable homogeneous chemical composition but shows similar dehydration microstructures. Based on these observations we argue that the formation of dispersed olivine grains as well as dehydration bands in our experiments does not depend on the local chemical composition.

### Dehydration due to induced heterogeneities

#### Thermal gradients

The steep Clapeyron slope of reaction 1 at our experimental pressure of 1.5 GPa suggests that already small temperature gradients can cause local dehydration (mechanism *d* in Fig. 10). The geometry of the sample assembly gives rise to a temperature gradient of up to 100 °C. In experiments equilibrated at 650 °C (thermocouple temperature), we expect the formation of anhydrous phases from reaction 1 primarily in the lower part of the capsule (Fig. 8), whereas the upper part should remain unaffected by dehydration. Both samples N_nd_650_m and Z_d_620_00 show enhanced dehydration in one half of the sample (supplementary material *F*), suggesting a contribution of the thermal gradient. Yet, independent of the thermocouple temperature (Table 1) we do observe dehydration bands in all experiments, except N_nd_650_m-KI, which cut the calculated potential temperature gradient at a high angle (Fig. 8d) and do not reflect the expected temperature distribution. Hence, thermal gradients present during the experiments may assist dehydration in general but are not responsible for the localized dehydration.

#### Mechanical driving potentials

We observed conjugate sets of dehydration bands as well as dispersed olivine formation associated with high-strain domains in samples subjected to a differential stress (Figs. 3 and 4). Additionally, we see dehydration products in hydrostatic samples N_nd_650_m and Z_nd_650_45. However, the latter experienced large differential stresses during initial compression due to a stuck piston (cf. *Methods* section), which caused significant permanent deformation of the sample observed as bulging of the capsule (supplementary material *C* and *F*). In sample N_nd_650_m dehydration products are observed in a set of conjugate bands (Fig. 5c-d). It is intriguing that most of the dehydration products formed in one half of the capsule, which may point to a contribution of the temperature gradient (cf. section *Thermal gradients*). However, such gradients cannot explain the steep inclination of the bands. It is likely that dehydration is influenced by stress perturbations caused by the high density of magnetite in this sample, which may serve as nucleation points for stress concentration during compaction (Baïsset et al. 2024). To the contrary, N_nd_650_m-KI, in which we aimed to minimize any remaining differential stresses by replacing the pressure medium with weaker KI, does not show signs of dehydration. Although temperature differences between our experiments were small (50 °C) and uncertainties in the measured thermocouple temperature do exists, the above discussed observations raise the question on the relationship between the mechanical responses to applied stress and mineral reactions (mechanism *e* in Fig. 10).

Chernak and Hirth (2010) performed similar co-axial deformation experiments on both core and powder samples and report the formation of through-going faults in serpentine experiments prior to dehydration at conditions of 400 °C to 550 °C, 1.5 GPa and strain rates of 10^− 5^ s^-1^. At the same conditions Auzende et al. (2015) reported cataclastic deformation in experiments. Such brittle behavior of antigorite can have important implications on the local phase relations: (i) Grain-size reduction through cataclasis along faults can increase the probability for nucleation and thereby increase the reaction kinetics (French et al. 2019; Burdette and Hirth 2020, 2022); and (ii) Shear heating along faults causes high temperatures at the slip plane and leads to dehydration. Microstructural observations in our samples indicate, however, that brittle deformation and associated processes are not responsible for the observed dehydration. N_nd_650_m-KI is the only sample with a micro-gouge (Fig. 2c). We did not apply a differential stress on this sample and cataclastic deformation probably happened during compression (i.e., before experimental target *PT*-conditions were reached). The formation of dehydration products within the micro-gouge is not observed. Other samples showing clear evidence for brittle deformation are N_nd_650_m and Z_d_620_90, exhibiting damage zones in the corners. Diffuse olivine formation is associated with these areas (Fig. 3a,b) but interestingly only in the adjacent domain between bent and kinked antigorite grains, while the most cataclastic part does not show dehydration. In samples showing dehydration bands related to through-going faults, we often observe anhydrous phases only on one side of the fault (Fig. 4a, b), whereas shear heating would cause a symmetric temperature gradient around the fault plane. We want to additionally emphasize here that shear heating strongly depends on the strain rate and very fast slip is required to induce dehydration in serpentinites.

Since bulging of the capsule is observed in almost all experiments (supplementary material *C*), we further explore the potential of visco-elasto-plastic deformation to cause dehydration in our experiments. While elastic strain might play a subordinate role in polycrystalline materials (de Ronde and Stünitz 2007), dislocations in individual antigorite crystals can increase the internal energy so that the energy barrier for the dehydration reactions is overstepped. Dispersed olivine formation is associated with high-strain domains close to damage zones and kink bands or between magnetite grains where stress localizes (Fig. 3). The dehydration bands often form combined shortening and extension structures (Fig. 4a,b). Such structures evolve when several (sub-)parallel deformation bands interfere with each other and result in domains with higher strain. Localized dehydration in such structures is also documented for example by Jung et al. (2004) and French et al. (2019). On a micro-scale, olivine formation in our samples is associated with bend and kinked antigorite grains (Figs. 2b and 5). The kernel average misorientations does not reveal significantly larger misorientation of antigorite within the dehydration bands (Fig. 6d). However, the observed relict antigorite grains might be those that are less affected by visco-elasto-plastic deformation. All together, these observations suggest that mechanical work during the visco-elasto-plastic deformation strongly influences the dehydration reaction and we discuss this hypothesis in the next section on the basis of a 2D numerical HMC model.

### Kinetics controlled by mechanical work rate: test with a 2D numerical HMC model

As described above, the conjugate set of dehydration bands observed in the experimental samples are approximately straight, oblique to the maximal shortening direction and have an extension much larger than the individual grains that compose them. The observations discussed above show that the hydrostatic effects such as temperature, as well as local mineralogical, chemical, and crystallographic heterogeneities do not provide a direct explanation for the dehydration of antigorite. Instead, we hypothesize that the generation of the anhydrous phases in the dehydration bands is caused by a kinetic process that is controlled by a mechanical quantity associated with shear bands forming during the deformation of the antigorite at high *PT*-conditions of the experiment. Such shear bands can be caused by plastic yielding, are oblique to the principal compression direction, and span across many antigorite grains during their initiation. This assumption is further in agreement with the lack of significant stress drops during the experiments (Fig. 1c).

A potential quantity to control the kinetics in a shear band is the mechanical work rate, *W*. The use of a scalar quantity is supported by the overall random orientation of olivine in the bands (Fig. 6c and supplementary material *E*), which suggests that no directed quantity is acting, such as a normal stress on a mineral boundary. Higher values of $\:W$ inside the shear bands cause faster kinetics inside the shear bands than outside (Fig. 9i - l). The increased kinetics causing the olivine + orthopyroxene formation by dehydration inside the shear bands is visible by increasing $\:{\rho\:}_{s}$ (Fig. 9m–p). Outside the shear bands the reaction progress is significantly slower and, consequently, $\:{\rho\:}_{s}$ remains approximately constant at the value corresponding to antigorite (Fig. 9e–h). The dehydration veins represent high-porosity pathways for fluids, which allow the released water to migrate along these veins to the sample boundaries. The performed simulation indicates that our hypothesis for the generation of olivine bands due to work rate-controlled kinetics is mechanically and thermodynamically feasible, in the sense that a HMC model can self-consistently predict dehydration bands. Self-consistent means here that the location and orientation of the dehydration bands is not prescribed in the model but controlled by $\:W$ that is calculated at each time step from the governing equations.

In rocks deforming by dislocation creep, the value of $\:W$ governs the energy stored in newly formed dislocations and the grain size reduction as described in the so-called paleowattmeter (e.g., Austin and Evans 2007). Dislocation creep can increase the dislocation density in the crystal grains and, hence, increase its internal energy and decrease the activation energy that controls the kinetic rate (Eqs. 2–3). Such processes have been inferred in studies where considerably faster reaction kinetics have been observed in deformed samples compared to undeformed ones (De Ronde and Stünitz 2007; Richter et al. 2016). Applied to our experiments, this may suggest that as a first step deformation is localized in shear bands, as indicated by the deformed capsules (supplementary material *C*). This is in contrast with previous experimental studies showing (semi-)brittle behavior at similar *PT* conditions (Chernak and Hirth 2010; Auzende et al. 2015). The slower strain rates in our experiments (~ 10^− 6^ s^− 1^), however, may explain the switch to more ductile behavior. A combination of deformation processes, including bending and kinking, grain size reduction and dislocation creep, help to overcome the energy barrier of the reaction, which then finally leads to local dehydration. In experiments that were heated to only 620 °C (thermocouple temperature), this extra energy added in form of the mechanical work might have been enough to locally move the reaction boundary and initiate dehydration. Upon dehydration, elevated porosity and transiently higher permeability emerge within the shear bands, while subsequent fluid migration and pore space collapse results in a partially dehydrated mineral assemblage with low porosity, as observed in our samples. We also observed dispersed olivine formation associated with high-strain domains and kink bands (Fig. 3). Our model suggests that strain accumulated locally in these regions caused the dehydration of antigorite. Furthermore, our model can explain why more dehydration products are observed around the diamond grains in Z_d_620_45-growth. The stiff diamond grains may have imposed local stress concentration on the antigorite grains. Finally, the model is in agreement with the slow reaction progress observed in our samples that reflects small overstep and thus slow reaction kinetics.

It is intriguing that the through-going faults that formed in some experiments are not centered in the dehydration bands and not all of the latter are associated with through-going faults. This suggests that these are two features resulting from axial compression but not intrinsically related to each other. If the cracking has formed during compression (i.e., before the *PT*-conditions of the experiment have been reached), only the kinetic effects of a surface increase by grain comminution may take effect. This effect has not been observed in our samples. Once the cracking occurs at the *PT*-conditions of the experiment the increased work term will be important. Furthermore, in some parts the slip plane evolved at the boundary between non-reacted antigorite and dehydration bands, suggesting that the rheology contrast could have been important for faulting.

In their deformation experiment performed at 625 °C and 1.5 GPa confining pressure, Chernak and Hirth (2010) reported a through-going fault without dehydration products. The authors used antigorite powder as a starting material for this experiment, which resulted in significantly less shear band formation. According to our experiments and numerical model, shear bands appear to be a key factor in driving the kinetics of the dehydration at «low» temperature. In contrast, the authors report distributed deformation for experiments run at 700 °C, which according to our model will lead to evenly distributed enhanced reaction kinetics. Diffuse olivine formation reported for these experiments, however, may also result from the high temperature above the thermal stability limit of antigorite rather than increased reaction kinetics due to mechanical effects. The authors also found prominent weakening associated with partial dehydration. In our experiments we observed that samples with 45° foliation were stronger compared to samples with 0° and 90° foliation (Fig. 1c). Although counterintuitive at first sight, slightly higher density of dehydration bands in the latter (Table 1, supplementary material *F*), may suggest weakening through progressing dehydration in agreement with numerical simulations by Baïsset et al. (2024), showing that localized dehydration may weaken serpentinites.

### From small-scale experiments to large-scale tectonics: implications for subduction zone phase relations

Local dehydration of serpentinites and formation of olivine-rich veins has been discussed for Erro Tobbio (Plümper et al. 2017), Val Malenco (Clément et al. 2019), Zermatt-Saas (Gilio et al. 2019; Kempf et al. 2020) and Cerro del Almirez (Jabaloy et al. 2022, Padron-Navarta et al. 2011). Although some of the reported vein-hosting rocks show evidence of ductile deformation, stress and strain are typically not considered as a potential trigger for the reaction. Our study now highlights the prominent role of ductile deformation on the phase relations, which has strong effects on the interpretation of mineral assemblages.

Several studies report ductile plastic behavior of natural serpentinites at elevated *PT* conditions (Auzende et al. 2015; Li et al. 2004; Jabaloy et al. 2022, Padron-Navarta et al. 2012). Such behavior and the associated increase in energy can lead to local dehydration and vein formation without the need of brittle events. The onset of dehydration may take place at lower *PT* conditions at non-hydrostatic conditions with respect to fully hydrostatic conditions, potentially leading to overestimation of metamorphic conditions. Finally, the formation of planar shear bands and local dehydration may produce fluid conduits. Similar to olivine-rich veins formed from other processes (Huber et al. 2022; Menzel et al. 2025), connected pore space in these zones has important implications for element redistribution, while the pore pressure affects the rheological behavior. Complete fluid drainage and resulting compaction, assisted through shear deformation, will eventually lead to an olivine-supported framework behaving more brittle at temperatures ≤ 700 °C with respect to pure serpentinites, i.e., leading to a ductile-brittle transition.

## Conclusions

We performed high-pressure deformation experiments at 620 °C, 650 °C and 670 °C, at 1.5 GPa and low strain rates in the order of 10^− 6^ s^− 1^. Equilibrium thermodynamic calculations suggest progressing formation of olivine through various reactions: at 620 °C local olivine formation is expected to form from magnetite (reaction 5) upon H_2_ influx; at 650 °C additional pervasive olivine is expected from reaction 1; and at 670 °C no antigorite should be left. In our samples, however, antigorite is the major phase at all conditions and only limited magnetite-related olivine formed. Localized olivine formation in dehydration bands and associated with high-strain domains and kink bands is observed in all samples that underwent deformation. While magnetite-related olivine requires the infiltration of external H_2_ and can thus be explained by variations in hydrogen flux, we could neither find a correlation between the location of olivine formation in bands and domains with minor phases, nor with antigorite composition, experimental temperature gradients or brittle deformation. The localization in narrow bands inclined by 30° to 45° with respect to the cylinder axis suggests a visco-elasto-plastic deformation mechanism. The HMC model support this hypothesis by the formation of shear bands during axial shortening, in which the mechanical work rate *W*, that is related to the energy stored in dislocations and strained crystals, is increased with respect to the overall sample. This adds extra energy to crystals, eventually causing dehydration in the shear bands. While antigorite survives in the bulk sample due to slow reaction kinetics, our experiments show that deformation can accelerate reaction kinetics and lead to localized dehydration.

Therefore, we suggest that the mechanical work rate *W* is a suitable mechanical quantity that can (i) control the reaction kinetics of antigorite, (ii) add extra energy that shifts the phase boundaries, and (iii) explain the observed oblique dehydration bands in the deformed antigorite.

With regards to subduction zones our results suggest that differential stresses, typically between 1 and 100 MPa, along plate interphases should not be neglected. Strain in these rocks can shift the stability limits of hydrous phases and cause localized fluid release. This has implications on equilibrium pressure and temperature estimates as well as fluid migration. Fluid conduits may form without the need of brittle fracturing. Adding stress- and strain-related parameters such as the mechanical work rate to models could help to better constrain dynamic processes in subduction zones.

## Supplementary Information

Below is the link to the electronic supplementary material.

Supplementary Material 1

## Data Availability

All data presented in this study can be found in the article, the online supplementary material as well as in the data repository 10.24416/UU01-MZ64R8.
