# Supplementary material for: Coupling antigorite deformation and dehydration in high-pressure experiments
Source: Contrib Mineral Petrol. 2025 Aug 23;180(9):64. doi: 10.1007/s00410-025-02255-z (PMC12373715; doi:10.1007/s00410-025-02255-z)
Supplement: Supplementary file 1 — Supplementary Material 1 [file 410_2025_2255_MOESM1_ESM.pdf]

Supplementary material to

COUPLING ANTIGORITE DEFORMATION AND DEHYDRATION IN HIGH-PRESSURE EXPERIMENTS

by Lisa Eberhard, Mattia Luca Mazzucchelli, Stefan Markus Schmalholz, Holger Stünitz, Ahmed Addad, Patrick Cordier, Oliver Plümper.

Submitted to *Contributions to Mineralogy and Petrology*

Table of contents

A - Starting material characterization

B - Temperature field in the experimental assembly

C - Recovered samples

D - Mineralogical and chemical composition of experimental samples

E - Crystal-orientation of olivine and antigorite

F - Orientation of dehydration bands

References

A - Starting material characterization

The chemical composition of serpentine in the starting material was quantified with electron microprobe analyses (EMPA) on thinsections. We measured the elemental concentration on representative areas of 1 cm x 1 cm (experimental sample size: 6.3 mm diameter and ~10 mm length). Data were acquired with a JEOL JXA-8530F microprobe running at 20 kV and 30 nA. We used a spot size of 8 µm, 30 ms dwell time and 8 µm step size. Several spot measurements in the mapped areas were acquired for map quantification. Resulting maps were quantified with XMapTools 4.3 (Lanari et al. 2019, 2024).

Figure S1 shows the quantified element maps of the massive serpentinite used as starting material (serpentinite from Linnajavri, Norway). Al-rich and Al-poor areas are clearly visible. Cr shows a heterogeneous distribution closely related to Al. Fe content is lower in regions with a higher Al content. We interpret this spatial element distribution as the initial mantle heterogeneity. Former orthopyroxene contained more Al and Cr but slightly less Fe with respect to olivine. The element distribution in the serpentinite inherited the chemical variability of the mantle, implying an overall negligible element transport during serpentinization.

Figure S2 shows the chemical composition of antigorite in the foliated serpentinite used as starting material (serpentinite from Zermatt, Switzerland). Antigorite has a remarkable homogeneous composition. Magnetite grains are aligned within the foliation. The serpentinite experienced high pressure and temperature (Li et al. 2004) during subduction and is strongly deformed. Metamorphic processes during subduction and obduction might have caused chemical homogenization.

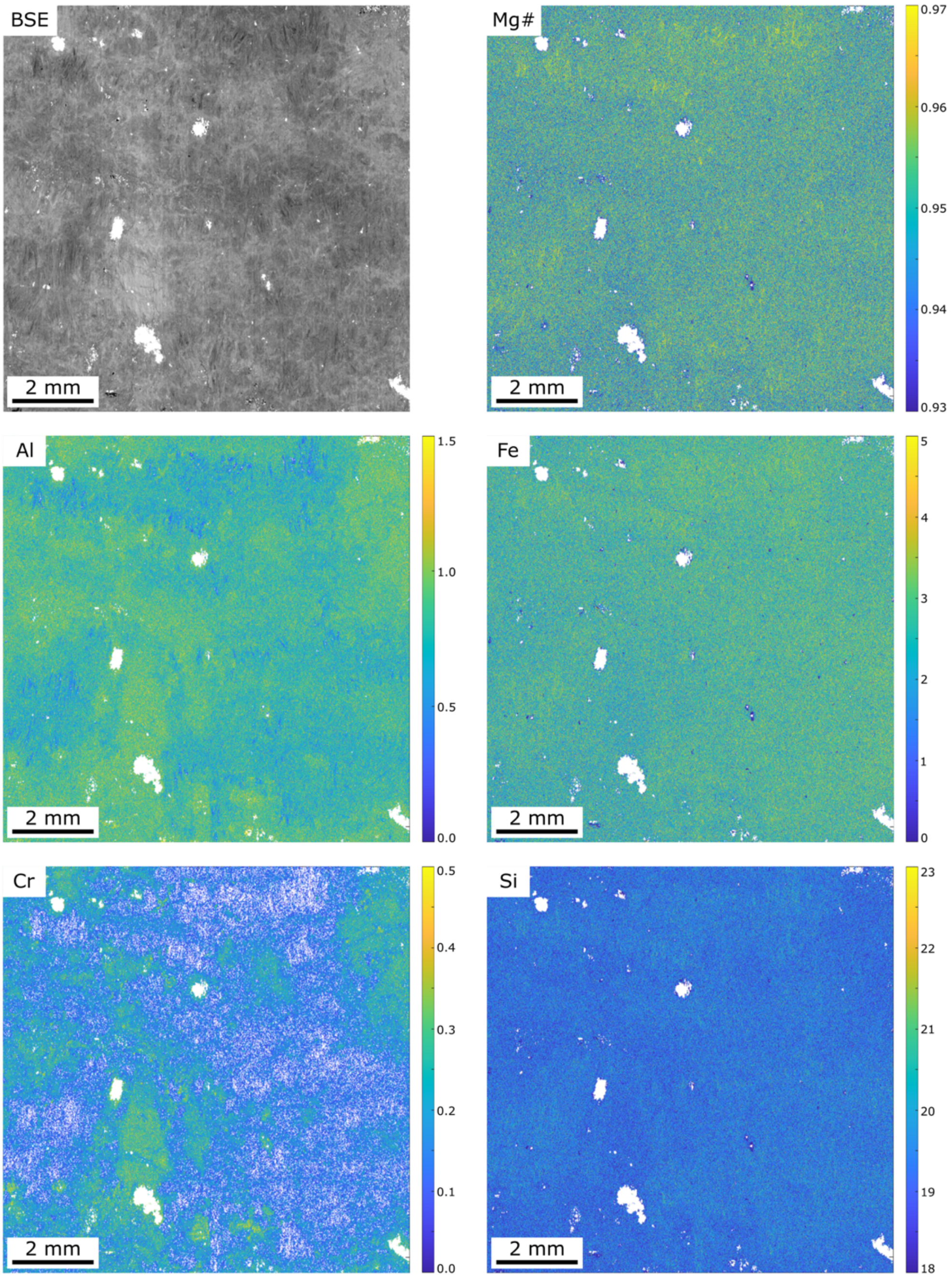

Figure S1: Back-scattered electron (BSE) map and chemical composition (wt. %) of antigorite in a massive serpentinite from Norway measured by EMPA. Maps were quantified with XMapsTools 4.3 (Lanari et al. 2019,

2024). Mg# is calculated as molar Mg/(Mg+Fe). White spots are magnetite grains (not quantified). Note the strong heterogeneous distribution of Al and Cr.

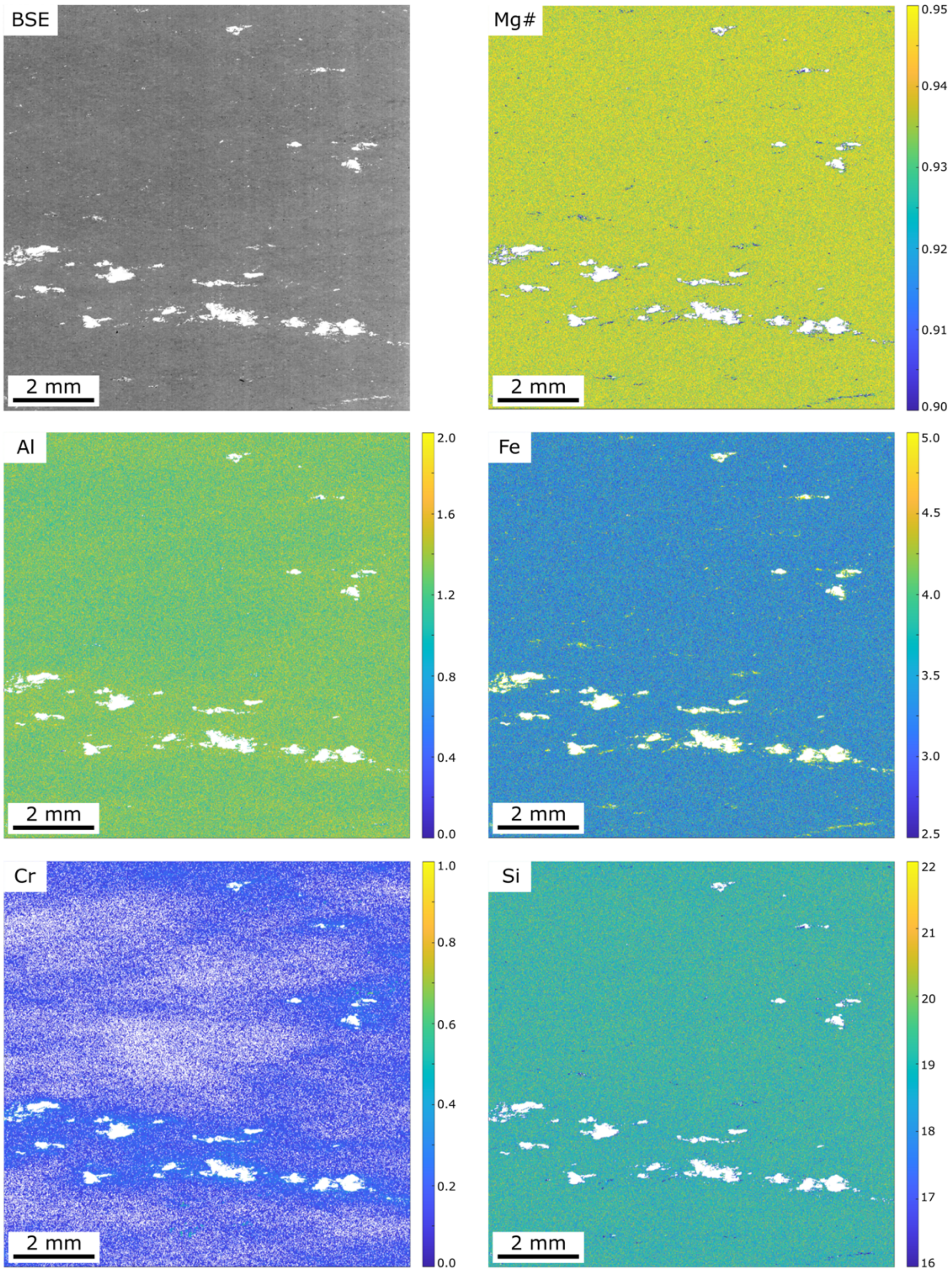

Figure S2: Back-scattered electron (BSE) map and chemical composition (wt. %) of antigorite in a foliated serpentinite from Zermatt measured by EMPA. Maps were quantified with XMapsTools 4.3 (Lanari et al. 2019, 2024). Mg# is calculated as molar Mg/(Mg+Fe). The antigorite is chemically homogeneous. White spots are magnetite grains (not quantified).

B - Temperature field in the experimental assembly

We estimated the temperature in the sample during the experiment with a numerical model initially published by Moarefvand et al. (2021). We solved the heat equation in cylindrical coordinates:

$$\rho\, C_p \frac{\partial T}{\partial t} = \frac{1}{r}\frac{\partial}{\partial r}\left(k\, r\, \frac{\partial T}{\partial r}\right) + \frac{\partial}{\partial y}\left(k \frac{\partial T}{\partial y}\right) + Q \qquad \text{(s1)}$$

Here ρ is the density at reference temperature of 20 °C, C_p is the heat capacity, k the thermal conductivity, T the temperature, Q the heat production, and r and y the cylindrical coordinates. Due to the axial symmetry of the sample, we performed the simulation on a half model to speed up the computation. The initial temperature was set to 19 °C, being the temperature of the cooling water. The temperature at the upper, lower and outer boundary was fixed to 19 °C, and the inner boundary was set to zero flux. Heat is generated only in the graphite. Heat capacity and thermal conductivity of each material are summarized in table S1. C_p and k are not pressure dependent and we also did not account for the compressibility and thermal expansion of the materials, i.e., constant ρ. However, preliminary simulations showed that these simplifications have limited effect on the final temperature field.

Table S1: Physical properties of each material used in the numerical simulation. References as followed: 1 Arblaster (2016); 2 Powell et al. (1966); 3 Chen et al. (2004); 4 Bauer & Urquhart (2016); 5 Krupka et al. (1979); 6 Carte (1955); 7 Holland & Powell (2011); 8 Hernlund et al. (2006); 9 Osako et al. (2010); 10 Holland & Powell (2011); 11 Kanamori et al. (1968) ; 12 Powell et al. (1966); 13 Holland & Powell (2011); 14 Gronvold et al. (1988); 15 Williams (1998); 16 Ho et al. (1968); 17 Wattanasarn & Seetawan (2013); 18 Slifka et al. (1998); 19 Seville (1975); 20 Hernlund et al. (2006).

material	density [kg / m^3]	heat capacity [J / (kg K)]		thermal conductivity [W / (m K)]		ref.
	ρ	C_p0	$C_p(T)$	$k0$	$k(T)$	
steel	8000	469.32	$-9\text{x}10^{-5}\, T^2 + 3.075\text{x}10^{-1}\, T + 386.95$	21.9	$2.16\text{x}10^{-2}\, T + 15.5$	1, 2
salt	2170	871.7	$0.1420\, T + 830.0821$	4.9	$1.10\text{x}10^{-11}\, T^4 - 4.51\text{x}10^{-8}\, T^3 + 7.02\text{x}10^{-5}\, T^2 - 5.11\text{x}10^{-2}\, T + 16.6$	3, 4
pyrophyllite	2840	782.35	$6.43\text{x}10^2 \log(T) - 2.87\text{x}10^3$	3.6	$2.07\text{x}10^{-12}\, T^4 - 9.49\text{x}10^{-9}\, T^3 + 1.66\text{x}10^{-5}\, T^2 - 1.41\text{x}10^{-2}\, T + 6.5$	5, 6

graphite	2200	685	$-1.49\text{x}10^{-10}\ T^4 + 1.16\text{x}10^{-6}\ T^3 - 3.39\text{x}10^{-3}\ T^2 + 4.58\ T - 394$	109.84	$-4.62\text{x}10^{-9}\ T^3 + 4.42\text{x}10^{-5}\ T^2 - 13.9\ T + 156$	7, 8
serpentine	2600	958.9	$2.58\text{x}10^{-6}\ T^3 - 5.81\text{x}10^{-3}\ T^2 + 4.83\ T - 22.4$	2.61	$2.72 - 3.78\text{x}^{-4}\ T$	9
alumina	3950	803	$-1.16\text{x}10^{-9}\ T^4 + 4.87\text{x}10^{-6}\ T^3 - 7.56\text{x}10^{-3}\ T^2 + 5.33\ T - 274$	37.19	$4.10\text{x}10^{-11}\ T^4 - 1.90\text{x}10^{-7}\ T^3 + 3.33\text{x}10^{-4}\ T^2 - 2.69\text{x}10^{-1}\ T + 91.9$	10, 11
copper	8960	385.4	$1.07\text{x}10^{-1}\ T + 352$	401	$-6.77\text{x}10^{-2}\ \text{T} + 423$	12, 13
W-carbide	15630	165.98	$4.45\text{x}10^{-7}\ T^3 - 9.47\text{x}10^{-4}\ T^2 + 6.85\text{x}10^{-1} T + 24.8$	57.7	$-1.38\text{x}10^{-2}\ T + 61.7$	14, 15
lead	11300	130	$3.22\text{x}10^{-2}\ T + 120.71$	35.7	$-1.59\text{x}10^{-2}\ T + 40$	16
MgO	3550	914.38	$1.182\text{x}10^3 + 0.142\ T - 2.182\text{x}10^5\ T^2 + 2.6075\text{x}10\text{-}^9\ T^3 - 26.3\text{x}10^6\ T^{-2}$	43.22	$86.8669 - 0.1921\ T + 1.6082\text{x}10^{-4}\ T^2 - 4.6562\text{x}10^{-8}\ T^3$	17, 18
Pt	21450	131.065	$126.5775 + 0.0257\ T - 2.6117\text{x}10^5\ T^{-2}$	70	$9.39\text{x}10^6\ T^2 - 4.38\text{x}10^{-3}\ T + 72.4$	19, 20

C - Recovered samples

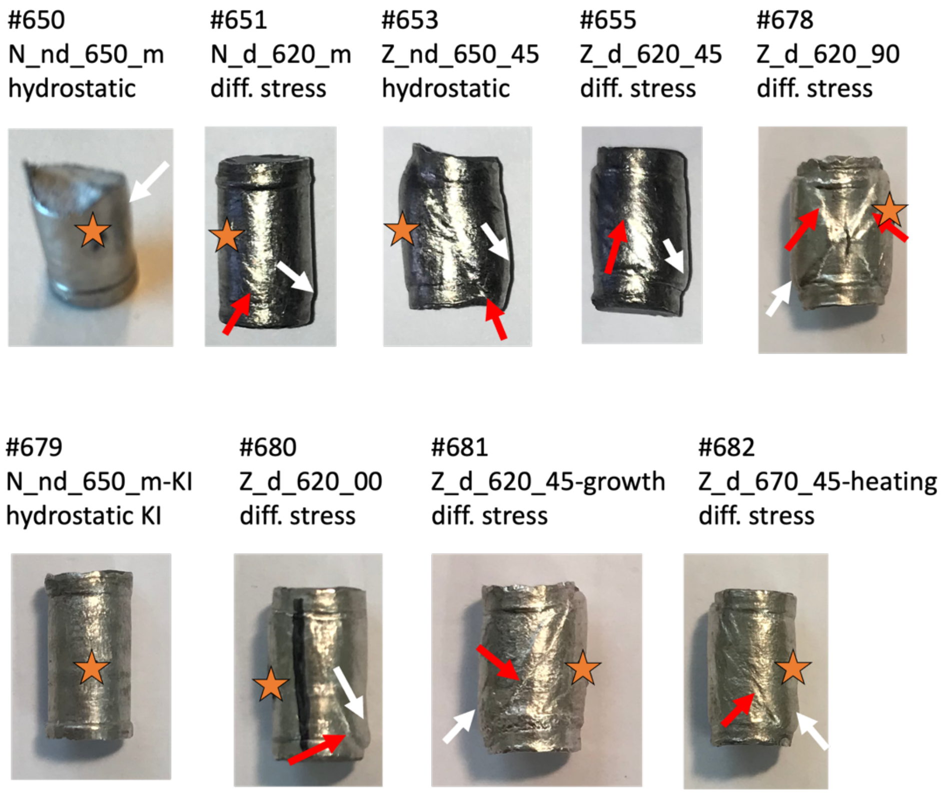

Figure S3: Recovered sample capsules showing bulging (white arrows) and shear bands (red arrows). Stars mark the position of the thermocouple, which typically left an imprint in the capsule, except for sample Z_d_620_45. Experimental conditions are given in table 1 of the main text.

D - Mineralogical and chemical composition of experimental samples

Qualitative phase identification was done by energy-dispersive X-ray (EDX) analyses. A Zeiss Gemini 450 SEM, operated at 15 kV and 1 - 2 nA was used to acquire EDX maps and points. Examples for phase identification are shown in figures S4 and S5. Note that it was not possible to identify individual phases in dehydration bands due to the fine-grained nature of this material. These phases were identified by transmission-electron microscopy (see main text and supplementary material *E* for methods and results).

The chemical composition of serpentine in two representative samples (N_nd_650_m, Z_nd_650_45) was quantified with a JEOL JXA-8530F microprobe running at 20 kV and 30 nA. A spot size of 7 µm and dwell time of 30 ms was used for acquisition. Resulting maps (Fig. S6 and S7) were quantified with XMapTools 4.3 (Lanari et al. 2019, 2024). Note that it was not possible to segment the fine-grained olivine from the serpentine matrix. Consequently, some Fe-rich and/or Mg-rich rims around magnetite and in bands crosscutting the sample are labelled here as serpentine but represent intergrown fine-grained antigorite+olivine±orthopyroxene. The mineralogy of these aggregates was determined by transmission-electron microscopy (c.f. main text and supplementary material *E*).

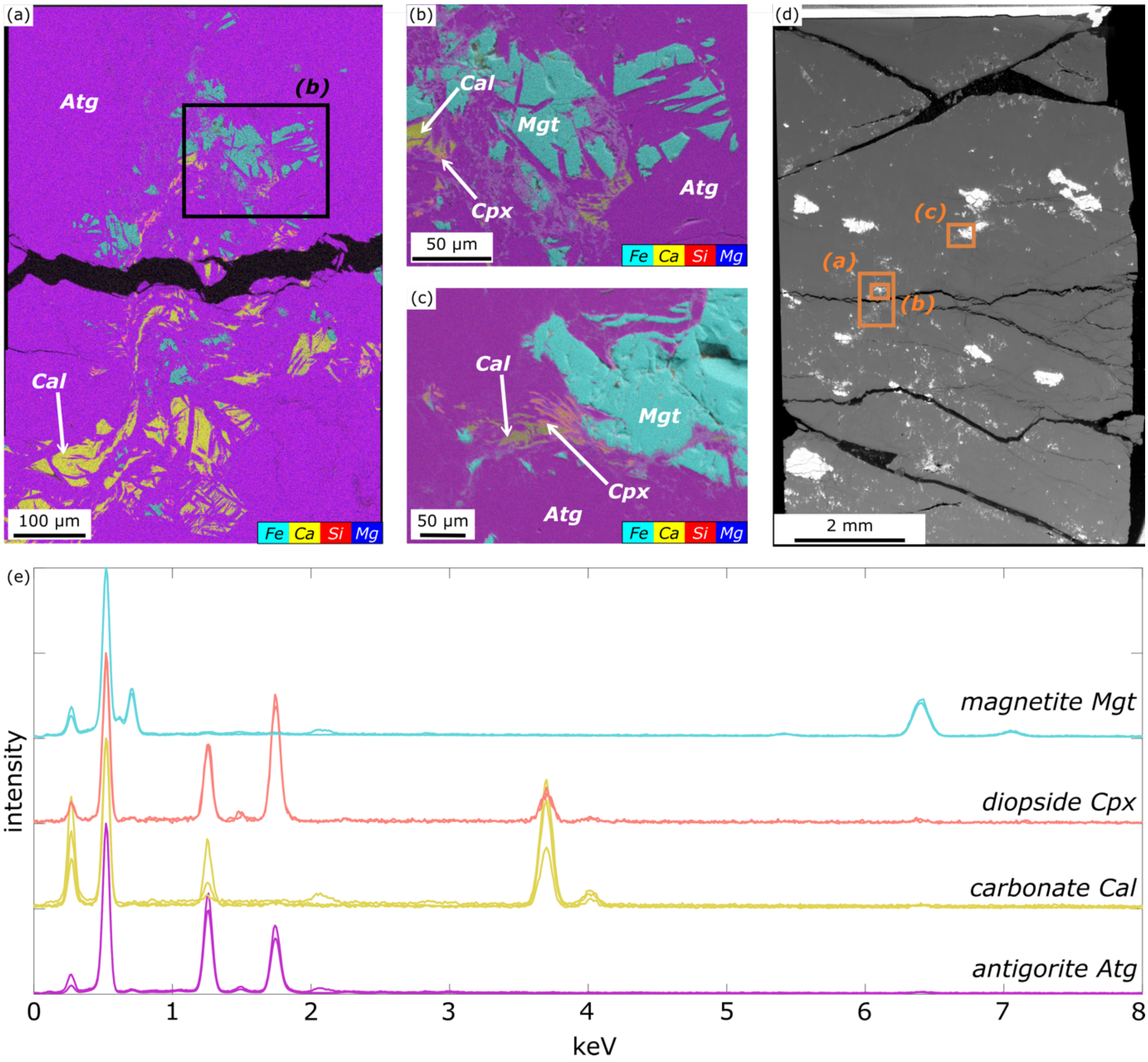

Figure S4: a - c) EDX maps of sample N_nd_650_m. d) BSE map showing the location of EDX maps in the sample. e) EDX spectra of phases highlighted in maps.

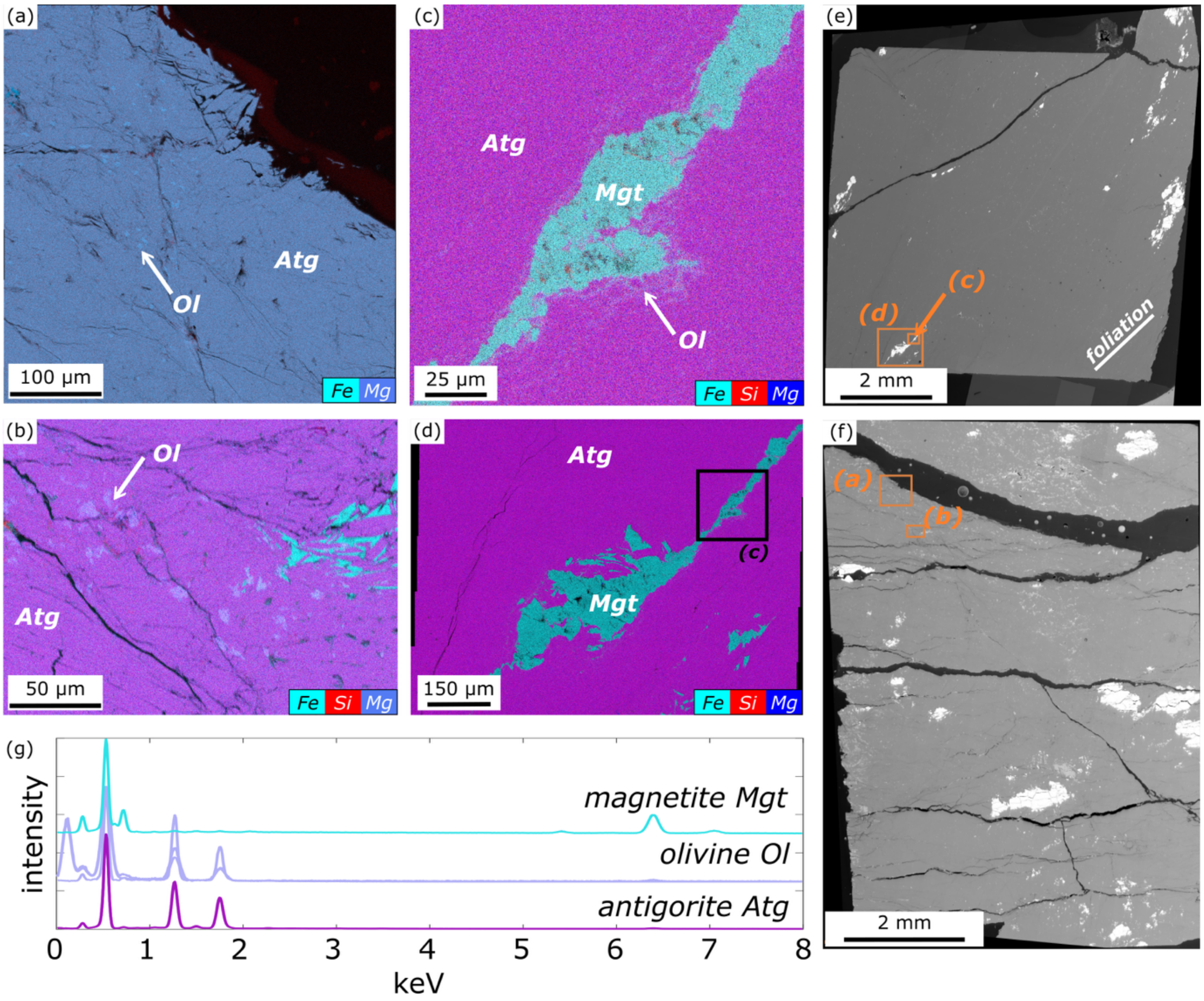

Figure S5: EDX maps of sample N_d_620_m (a, b) and Z_nd_650_45 (c, d). e-f) BSE maps of sample Z_nd_650_45 (e) and N_d_620_m (f) showing the location of the EDX maps. g) EDX spectra from phases highlighted in EDX maps. Note that olivine is fine-grained and the spectra often show mixtures of antigorite+olivine±orthopyroxene.

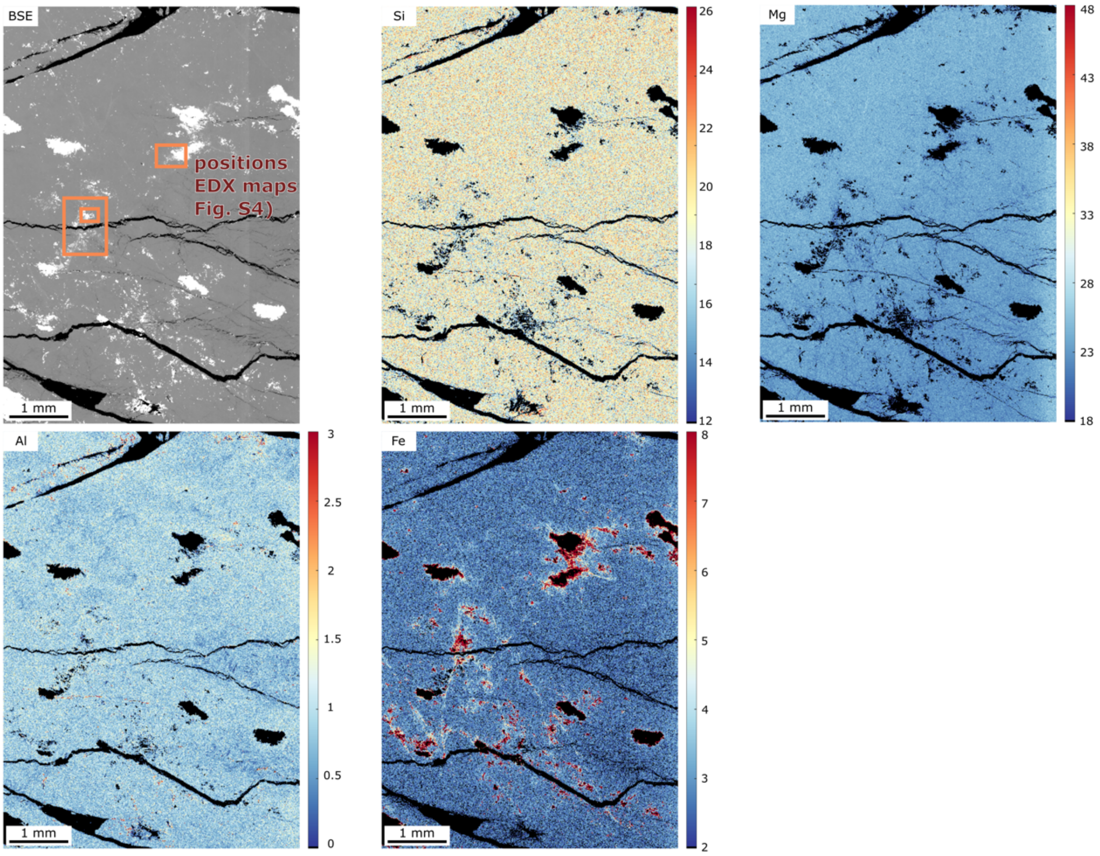

Figure S6: Back-scattered electron (BSE) map and chemical composition of antigorite in sample N_nd_650_m. Maps were quantified with XMapsTools 4.3 (Lanari et al. 2019, 2024). Black and white areas are epoxy, carbonate and magnetite (not quantified).

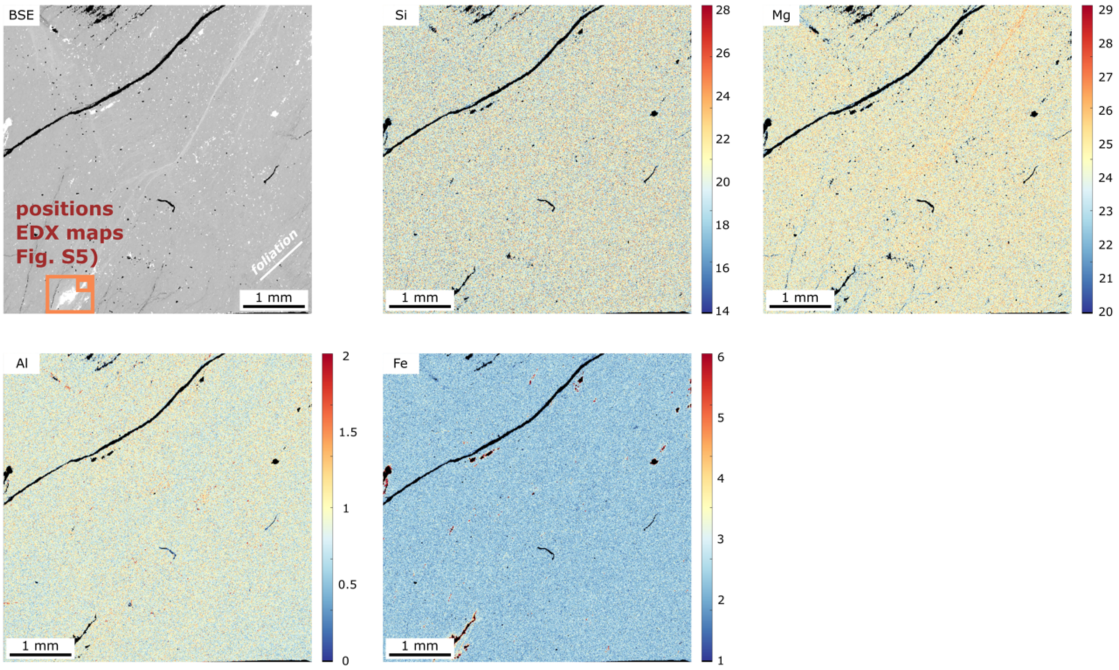

Figure S7: Back-scattered electron (BSE) map and chemical composition of antigorite in sample Z_nd_650_45. Maps were quantified with XMapsTools 4.3 (Lanari et al. 2019, 2024). Black and white areas are epoxy and magnetite (not quantified).

E – Crystal-orientation of olivine and antigorite

Crystal-orientation of antigorite and olivine within dehydration bands was determined on a selected area in a TEM foil prepared from sample Z_d_620_90 (Fig. S8a; c.f. main text for foil preparation), using automated crystal-orientation mapping technique (ACOM-TEM). Space groups for antigorite and olivine were Pm and Pbnm, respectively, orthopyroxene was not detected in the respective map area. Orientation data were acquired with NanoMEGAS ASTAR system on a FEI Tecnai G2-20 twin at the electron microscopy facility of the Advanced Characterization Platform of the Chevreul Institute in Lille. Beam conditions were 200 kV with a nominal probe diameter of 5 nm, a step size of 10 nm x 10 nm and a precession angle of 1°. Data reduction was done with the MATLAB toolbox MTEX (Bachmann et al. 2010). The thickness of the foil (~200 nm) caused difficulties in grain reconstruction of the platy antigorite, whereas grain reconstruction for olivine revealed good results. Consequently, the orientation distribution functions were calculated for olivine only. Olivine grains within the dehydration bands show a very weak crystal preferred orientation (CPO), with the b axis perpendicular to the compression axis (Fig. S8b).

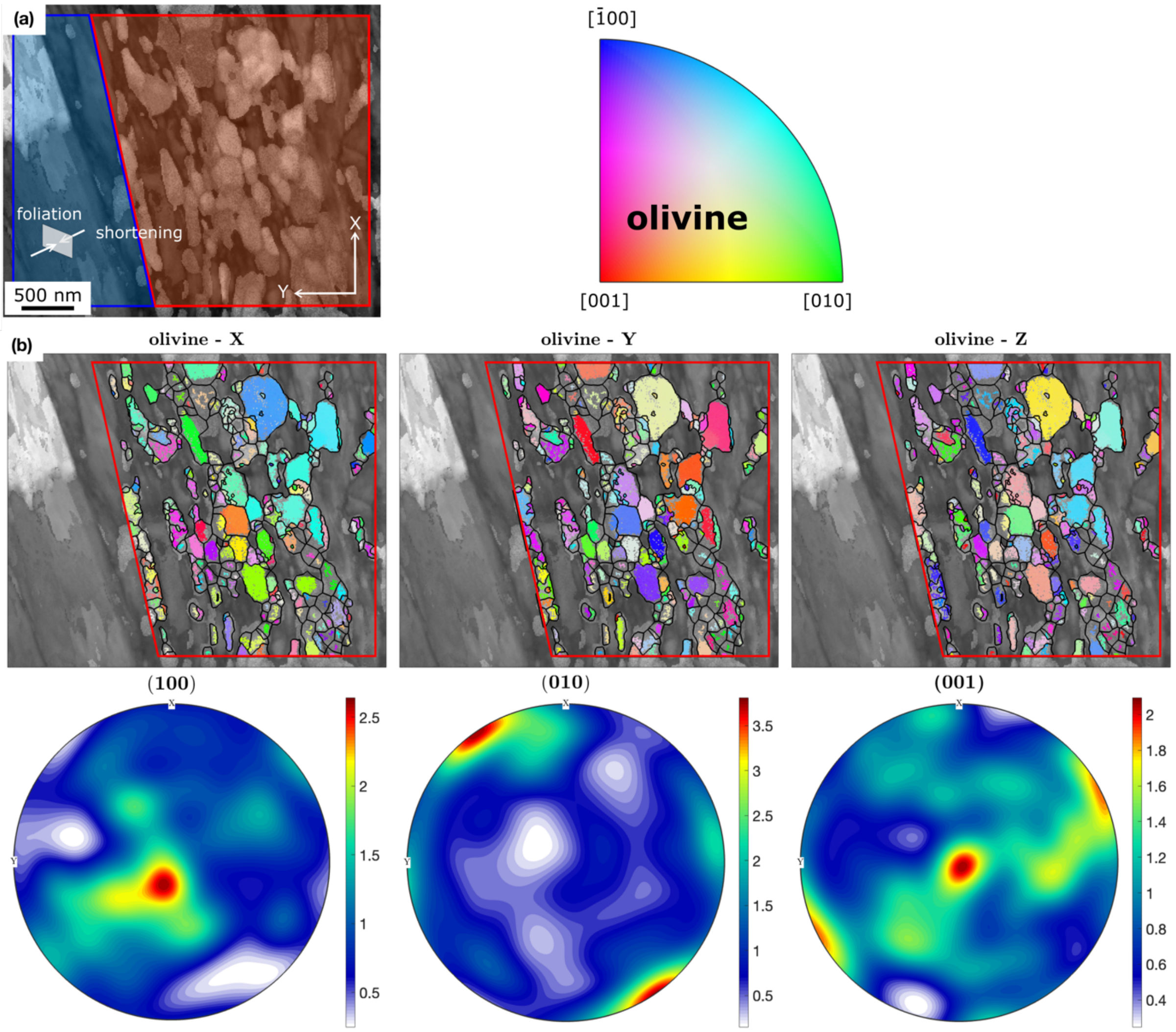

Figure S8: ACOM-TEM analyses. a) TEM image of a thin foil prepared from sample Z_d_620_90, showing the location of the acquired orientation map. The map is divided into a non-dehydrated (blue) and a dehydrated (red) domain. Orientations were obtained for olivine (space group Pbnm) in the dehydrated domain. b) IPF color of olivine and corresponding pole figures (one point per grain).

F – Orientation of dehydration bands

Electron back-scattered (BSE) maps of run products (high-resolution images can be found in the data repository: https://doi.org/10.24416/UU01-MZ64R8), manually extracted locations of dehydration reaction products (red: magnetite for orientation, cyan: primary calcite and diopside, purple: olivine and diopside formed from magnetite-antigorite and calcite-antigorite reaction, black lines: dehydration bands) and orientation of dehydration bands plotted with FracPaq (Healy et al. 2017).

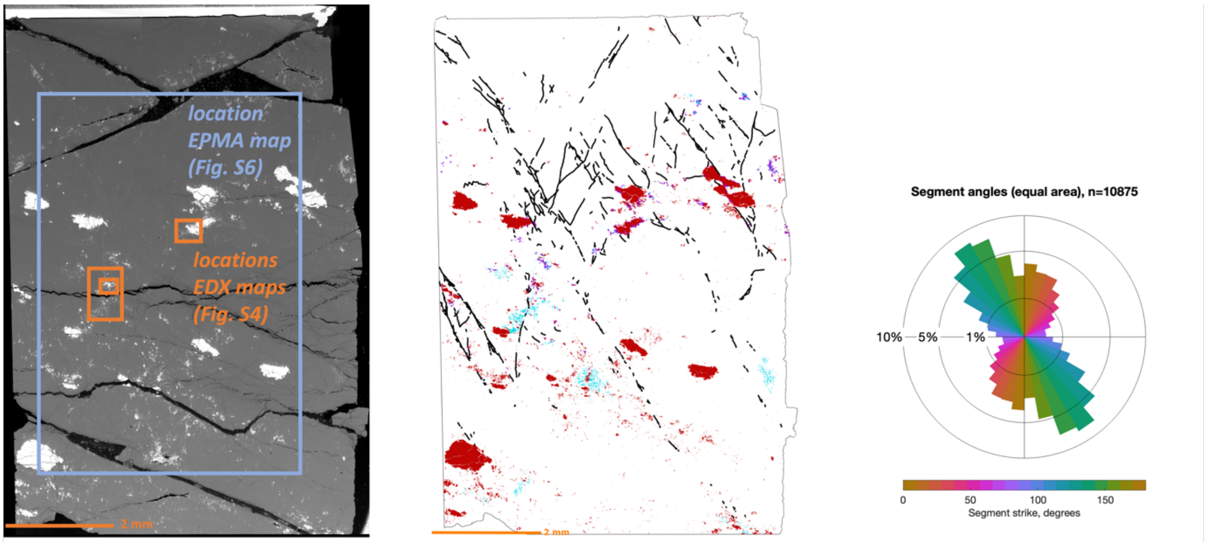

#650 - isotropic - 650 °C - 1.5 GPa confining pressure - no differential stress

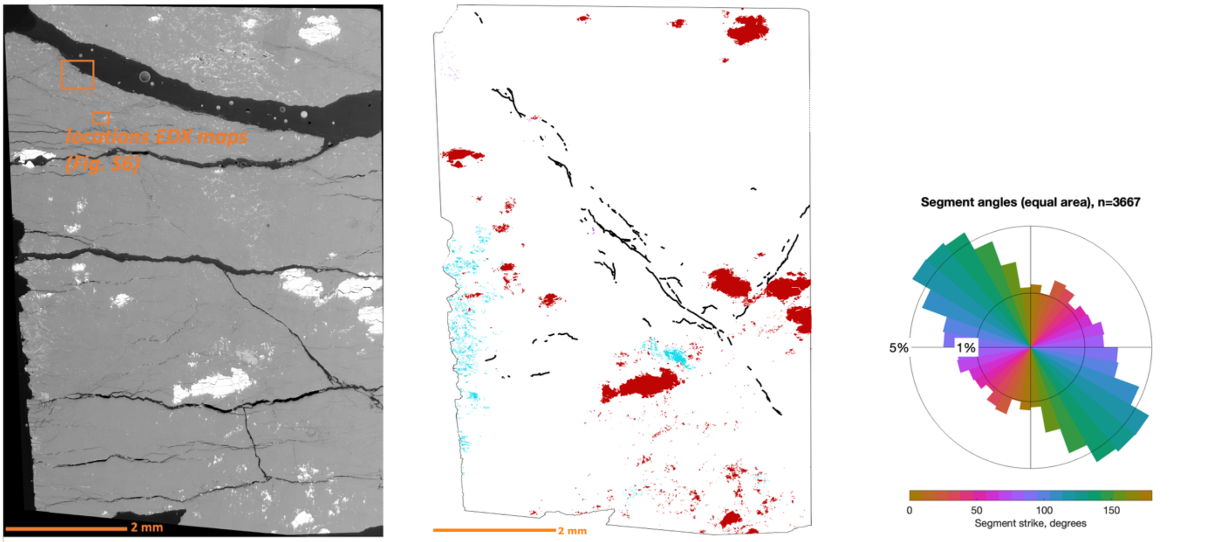

#651 - isotropic - 620 °C - 1.5 GPa confining pressure - 720 MPa differential stress

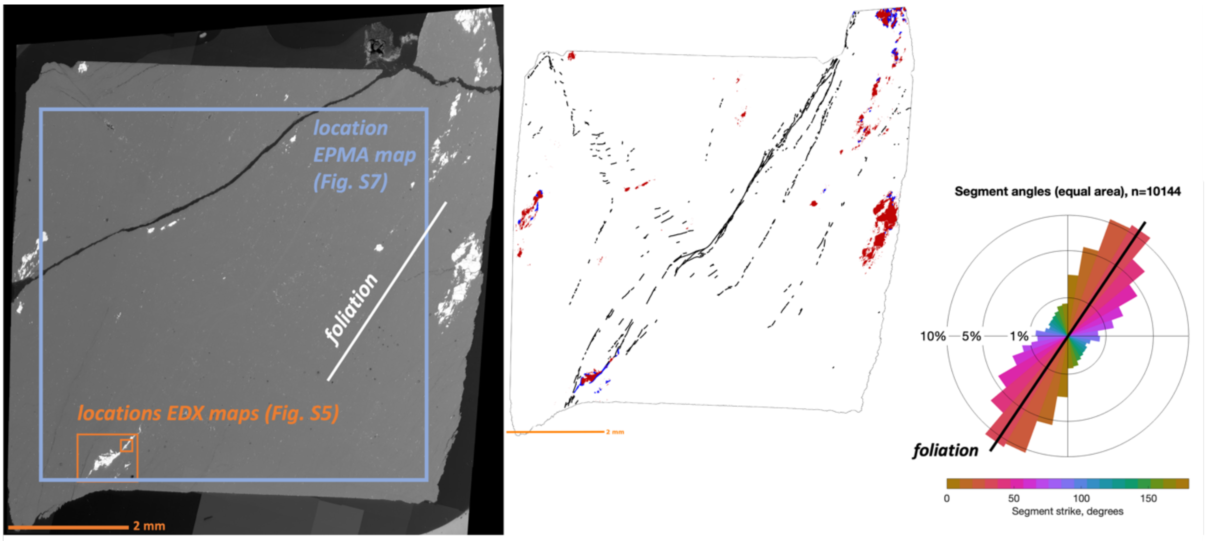

#653 - foliated (45°) - 650 °C - 1.5 GPa confining pressure - no differential stress - pre-strained (c.f. methods section in main text)

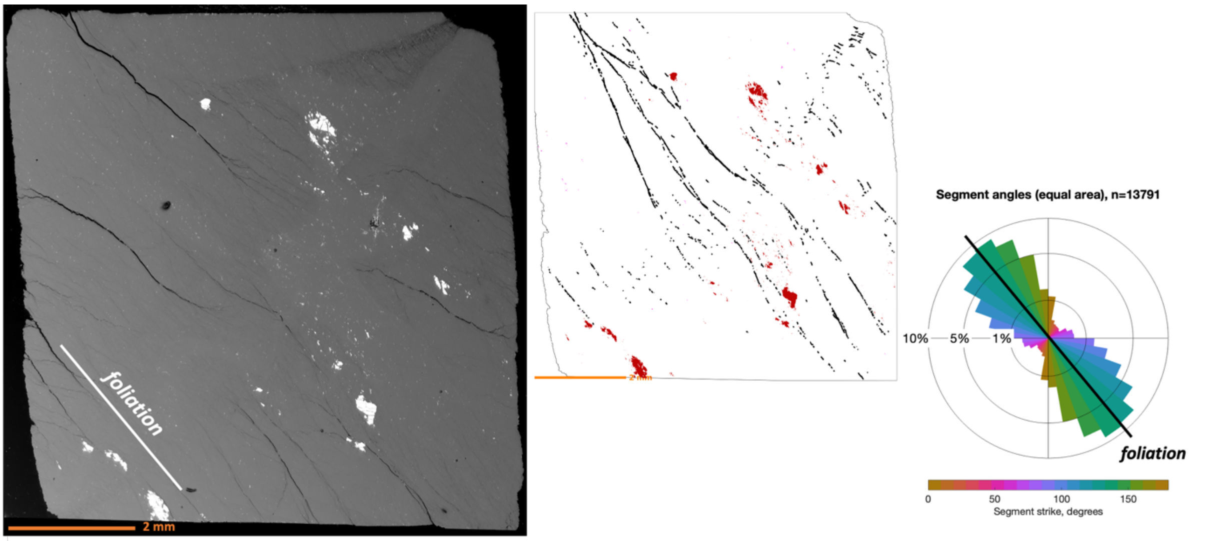

#655 - foliated (45°) - 620 °C - 1.5 GPa confining pressure - 598 MPa differential stress

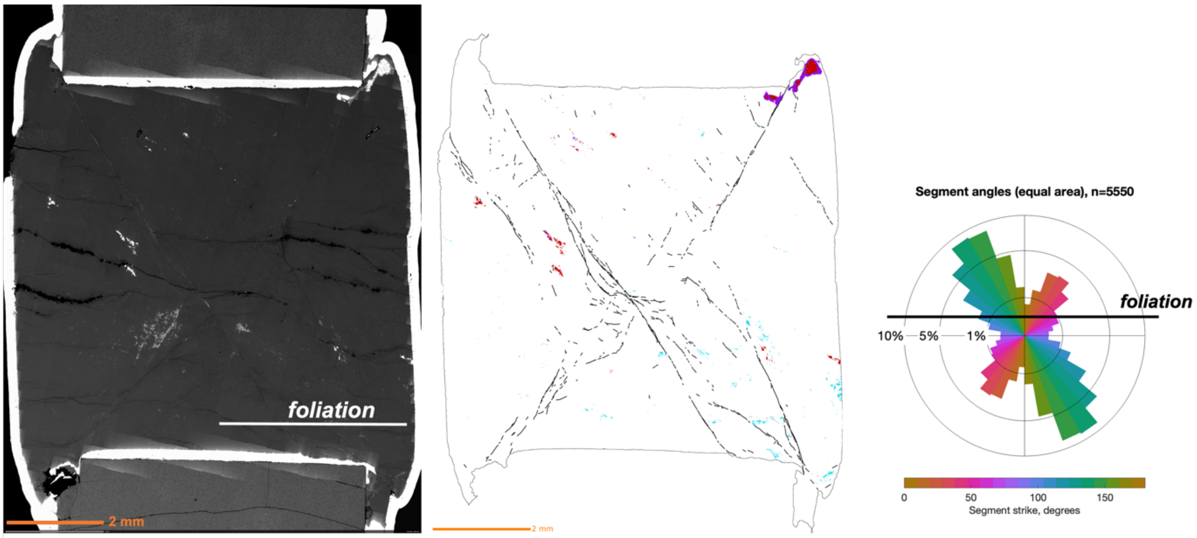

#678 - foliated (90°) - 620 °C - 1.5 GPa confining pressure - 370 MPa differential stress

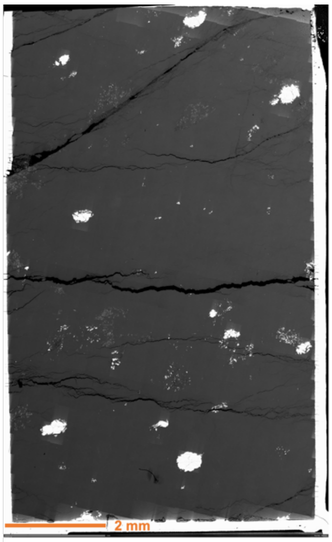

#679 - isotropic - 650 °C - 1.5 GPa confining pressure - no differential stress

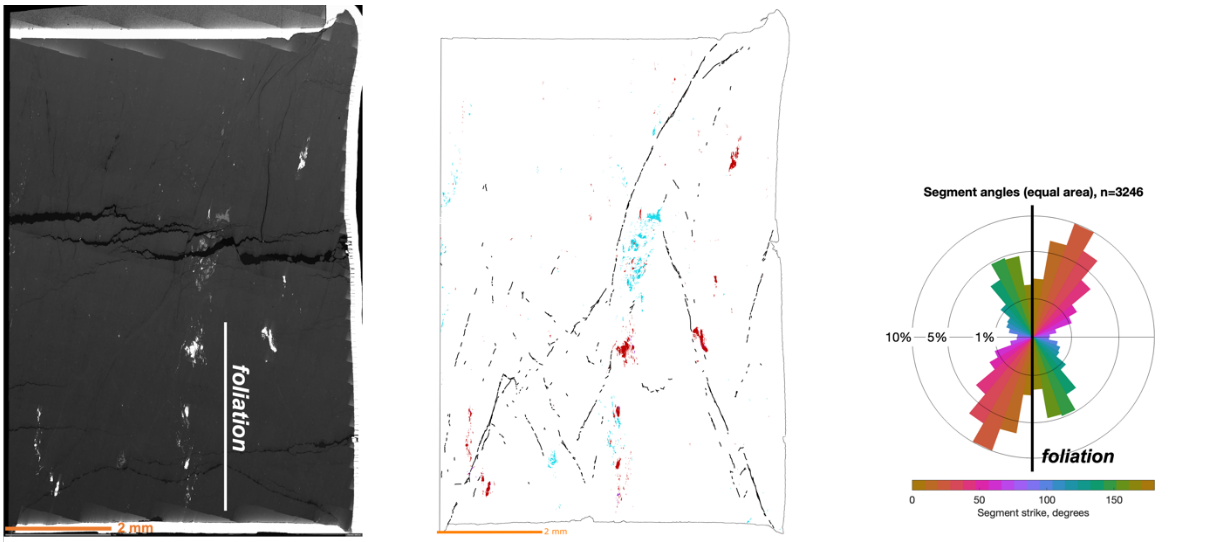

#680 - foliated (0°) - 620 °C - 1.5 GPa confining pressure - 549 MPa differential stress

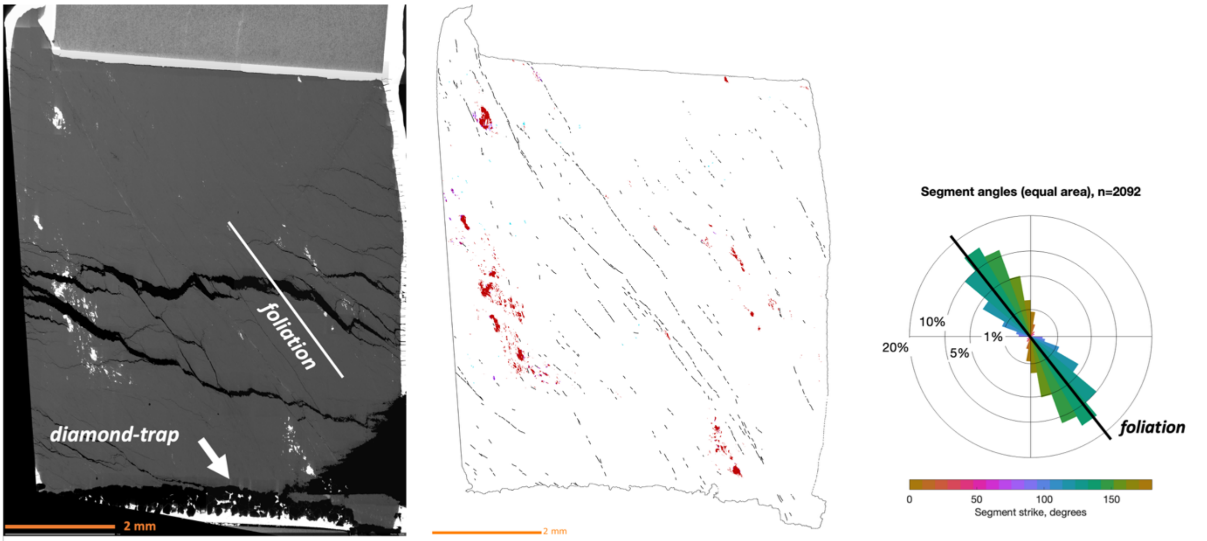

#681 - foliated (45°) - 620 °C - 1.5 GPa confining pressure - 695 MPa differential stress - diamond-trap

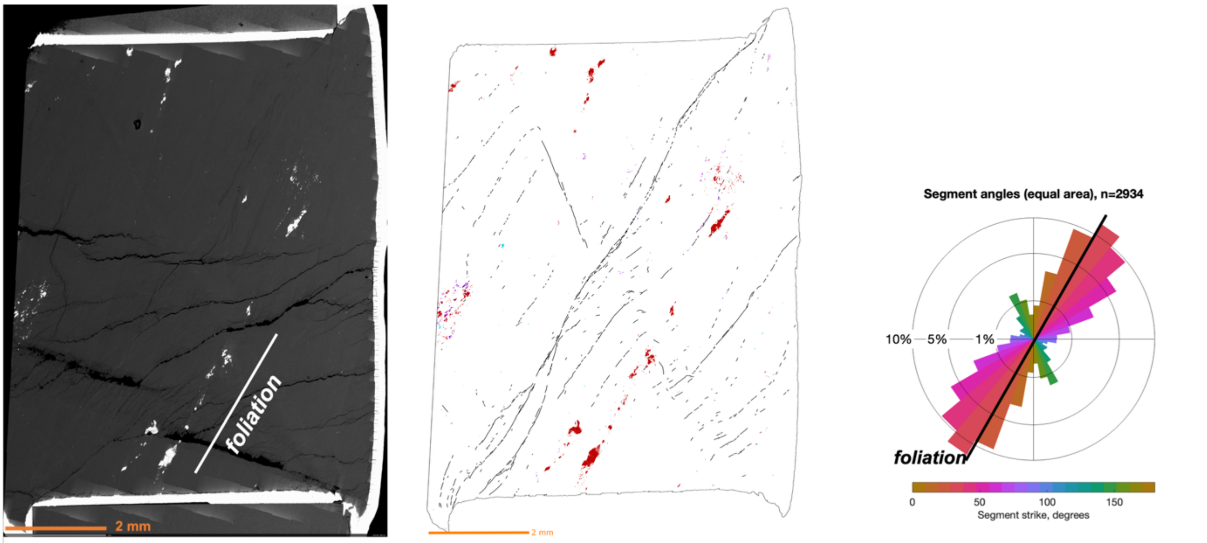

#682 - foliated (45°) - 620 - 670 °C - 1.5 GPa confining pressure - 601 MPa differential stress - heated during run

References

Arblaster JW (2016) Thermodynamic Properties of Gold. J Phase Equilib Diffus 37:229–245

Bachmann F, Hielscher R, Schaeben H (2010) Texture analysis with MTEX- Free and open source software toolbox. Solid State Phenom 160:63–68

Bauer S, Urquhart A (2016) Thermal and physical properties of reconsolidated crushed rock salt as a function of porosity and temperature. Acta Geotech 11:913–924

Carte AE (1955) Thermal constants of pyrophyllite and their change on heating. Br J Appl Phys 6:326–328

Chen Q, Cai L, Duan S, Chen D (2004) Molecular dynamics simulation of thermodynamic properties of NaCl at high pressures and temperatures. J Phys Chem Solids 65:1077–1081

Gronvold F, Stolen S, Westrum EF, Labban AK, Uhrenius B (1988) Heat Capacity and Thermodynamic Properties of Ditungsten Carbide, W_2C_{1-x}, from 10 to 1000 K. Thermochim Acta 129:115–125

Healy D, Rizzo RE, Cornwell DG, Farrell NJC, Watkins H, Timms NE, Gomez-Rivas E, Smith M (2017) FracPaQ: A MATLABTM toolbox for the quantification of fracture patterns. J Struct Geol 95:1–16

Hernlund J, Leinenweber K, Locke D, Tyburczy JA (2006) A numerical model for steady-state temperature distributions in solid-medium high-pressure cell assemblies. Am Min 91:295–305

Ho CY, Powell RW, Liley PE (1968) Thermal conductivity of selected materials, part 2. National Institute of Standards and Technology, Gaithersburg, MD

Holland TJB, Powell R (2011) An improved and extended internally consistent thermodynamic dataset for phases of petrological interest, involving a new equation of state for solids. J Metamorph Geol 29:333–383

Kanamori H, Fuji N, Mizutani H (1968) Thermal diffusivity measurement of rock-forming minerals from 300 ° to 1100 °K. J Geophys Res 73(2):595-605

Krupka KM, Robie RA, Hemmingway BS (1979) High-temperature heat capacities of corundum, periclase, anorthite, $CaAl_2Si_2O_8$ glass, muscovite, pyrophyllite, $KAlSi_3O_8$ glass, grossular, and $NaAlSi_3O_8$ glass. Am Min 64:86–101

Lanari P, Vho A, Bovay T, Airaghi L, Centrella S (2019) Quantitative compositional mapping of mineral phases by electron probe micro-analyser. Geological Society of London, Special Publications, 478, 39-63

Lanari P, Markmann T, Laughton J, Tedeschi M (2024). XMapTools 4.3 (v4.3). Zenodo. https://doi.org/10.5281/zenodo.10509537

Li XP, Rahn M, Bucher K (2004) Serpentinites of the Zermatt-Saas ophiolite complex and their texture evolution. J Metamorph Geol 22:159–177

Moarefvand A, Gasc J, Fauconnier J, Baïsset M, Burdette E, Labrousse L, Schubnel A. (2021) A new generation Griggs apparatus with active acoustic monitoring. Tectonophysics 816:229032

Osako M, Yoneda A, Ito E (2010) Thermal diffusivity, thermal conductivity and heat capacity of serpentine (antigorite) under high pressure. Phys Earth Planet Inter 183:229–233

Powell RW, Ho CY, Liley PE (1966) Thermal conductivity of selected materials. National Institute of Standards and Technology, Gaithersburg, MD

Seville AH (1975). The heat capacity of platinum at high temperatures. J Chem Thermodyn 7:383–387

Slifka AJ, Filla BJ, Phelps JM (1998) Thermal Conductivity of Magnesium Oxide from Absolute, Steady-State Measurements. J Res Natl Inst Stand Technol 103:357–363

Wattanasarn H, Seetawan T (2013) Studies thermophysical properties of MgO by first principle simulation. Adv Mat Res 802:139–143

Williams WS (1998) The Thermal Conductivity of Metallic Ceramics. JOM 50:62–66
